# Early life exposure to vitamin D deficiency impairs molecular mechanisms that regulate liver cholesterol biosynthesis, energy metabolism, inflammation, and detoxification

**DOI:** 10.3389/fendo.2024.1335855

**Published:** 2024-05-10

**Authors:** Megan M. Knuth, Jing Xue, Marwa Elnagheeb, Raad Z. Gharaibeh, Sarah A. Schoenrock, Susan McRitchie, Cory Brouwer, Susan J. Sumner, Lisa Tarantino, William Valdar, R. Scott Rector, Jeremy M. Simon, Folami Ideraabdullah

**Affiliations:** ^1^ Department of Genetics, School of Medicine, University of North Carolina at Chapel Hill, Chapel Hill, NC, United States; ^2^ Lineberger Comprehensive Cancer Center, University of North Carolina at Chapel Hill, Chapel Hill, NC, United States; ^3^ Nutrition Research Institute, University of North Carolina at Chapel Hill, Kannapolis, NC, United States; ^4^ Department of Medicine, Division of Gastroenterology, University of Florida, Gainesville, FL, United States; ^5^ Department of Molecular Genetics and Microbiology, University of Florida, Gainesville, FL, United States; ^6^ Department of Bioinformatics and Genomics, University of North Carolina at Charlotte, Charlotte, NC, United States; ^7^ University of North Carolina at Charlotte Bioinformatics Service Division, North Carolina Research Campus, Kannapolis, NC, United States; ^8^ Department of Nutrition, Gillings School of Public Health, University of North Carolina at Chapel Hill, Chapel Hill, NC, United States; ^9^ Division of Pharmacotherapy and Experimental Therapeutics, Eshelman School of Pharmacy, University of North Carolina at Chapel Hill, Chapel Hill, NC, United States; ^10^ Department of Psychiatry, School of Medicine, University of North Carolina at Chapel Hill, Chapel Hill, NC, United States; ^11^ Research Service, Harry S. Truman Memorial Veterans Medical Center, Columbia, MO, United States; ^12^ NextGen Precision Health, University of Missouri, Columbia, MO, United States; ^13^ Department of Nutrition and Exercise Physiology, University of Missouri, Columbia, MO, United States; ^14^ Division of Gastroenterology and Hepatology, Department of Medicine, University of Missouri, Columbia, MO, United States; ^15^ Neuroscience Center Bioinformatics Core, University of North Carolina at Chapel Hill, Chapel Hill, NC, United States

**Keywords:** vitamin D, DOHaD, liver disease, parental origin, susceptibility, omics

## Abstract

**Introduction:**

Emerging data suggests liver disease may be initiated during development when there is high genome plasticity and the molecular pathways supporting liver function are being developed.

**Methods:**

Here, we leveraged our Collaborative Cross mouse model of developmental vitamin D deficiency (DVD) to investigate the role of DVD in dysregulating the molecular mechanisms underlying liver disease. We defined the effects on the adult liver transcriptome and metabolome and examined the role of epigenetic dysregulation. Given that the parental origin of the genome (POG) influences response to DVD, we used our established POG model [POG1-(CC011xCC001)F1 and POG2-(CC001xCC011)F1] to identify interindividual differences.

**Results:**

We found that DVD altered the adult liver transcriptome, primarily downregulating genes controlling liver development, response to injury/infection (detoxification & inflammation), cholesterol biosynthesis, and energy production. In concordance with these transcriptional changes, we found that DVD decreased liver cell membrane-associated lipids (including cholesterol) and pentose phosphate pathway metabolites. Each POG also exhibited distinct responses. POG1 exhibited almost 2X more differentially expressed genes (DEGs) with effects indicative of increased energy utilization. This included upregulation of lipid and amino acid metabolism genes and increased intermediate lipid and amino acid metabolites, increased energy cofactors, and decreased energy substrates. POG2 exhibited broader downregulation of cholesterol biosynthesis genes with a metabolomics profile indicative of decreased energy utilization. Although DVD primarily caused loss of liver DNA methylation for both POGs, only one epimutation was shared, and POG2 had 6.5X more differentially methylated genes. Differential methylation was detected at DEGs regulating developmental processes such as amino acid transport (POG1) and cell growth & differentiation (e.g., Wnt & cadherin signaling, POG2).

**Conclusions:**

These findings implicate a novel role for maternal vitamin D in programming essential offspring liver functions that are dysregulated in liver disease. Importantly, impairment of these processes was not rescued by vitamin D treatment at weaning, suggesting these effects require preventative measures. Substantial differences in POG response to DVD demonstrate that the parental genomic context of exposure determines offspring susceptibility.

## 1 Introduction

Nonalcoholic fatty liver disease (NAFLD) is a metabolic disease characterized by excessive liver fat accumulation (fat content exceeds 5% of liver weight) and inflammation (1–3). NAFLD, associated with underlying metabolic syndrome, including type 2 diabetes, high blood pressure, elevated triglycerides and cholesterol, was recently renamed “Metabolic dysfunction-Associated Fatty Liver Disease” (MAFLD) (4). The worldwide prevalence of NAFLD is increasing annually, with recent (2020) rates of 25% among individuals aged 2-70+ (5–8). NAFLD alone can have severe health consequences, and when left untreated, NAFLD can progress into more severe end-stage liver disease such as hepatocellular carcinoma (HCC). HCC is one of the leading causes of cancer-related death worldwide (6, 7). Therefore, it is critical to elucidate the early causes and mechanisms underlying NAFLD.

The most marked characteristic of NAFLD is liver fat accumulation caused by metabolic dyshomeostasis (8), whereby energy production is disrupted in favor of energy storage. While metabolic dyshomeostasis can occur at any point in time, *in utero* development has long been recognized as a sensitized window of exposure (9) when offspring are particularly vulnerable to nutrient fluctuations (10, 11) that can alter this balance. For example, depletion of vitamin D levels during *in utero* development in Wistar rat models caused severe liver fat accumulation in adulthood (12). This effect was attributed to underlying mitochondrial dysfunction and abnormal liver lipid metabolism (12). Sprague-Dawley rat models also showed that maternal vitamin D deficiency during pregnancy is associated with offspring insulin resistance, a key regulator of energy storage in the liver (13). In this model, the insulin resistance was attributed to deficiency-induced upregulation of inflammatory cytokines in the liver and serum (13).

Chronic inflammation is another key characteristic of NAFLD (3), which when untreated left can progress to fibrosis and HCC (14, 15). In Sprague-Dawley rat models of developmental vitamin D deficiency (DVD), DVD induced elevated liver and pancreatic oxidative stress levels in adult offspring despite vitamin D repletion (16). Following a similar exposure model, Masako et al. found that DVD in C57BL/6J mouse models caused permanent changes in the proportions of inflammatory cells in the adult liver and expression of genes regulating lipid metabolism (17). One hypothesis is that vitamin D deficiency during *in utero* development creates an inflammatory state in the fetus. Supporting this hypothesis, vitamin D has been shown to regulate placental inflammation during human pregnancy (18) and in C57BL/6J mice, developmental vitamin D deficiency promotes infiltration and activation of liver macrophages (17). However, the role of DVD in NAFLD-related liver inflammation has not been directly investigated.

Severe maternal vitamin D deficiency in humans during pregnancy impairs fetal growth and development, causing low birth weight and small for gestational age (SGA) (19, 20). Both low birth weight and SGA are recognized risk factors for NAFLD, especially under conditions of rapid postnatal weight gain (14, 15). This is in part due to underlying disruptions in metabolic-endocrine maintenance (21, 22), making the offspring more susceptible to liver disease outcomes.

Previously, we found that DVD exposure in Collaborative Cross (CC) mice caused increased adult body weight and fat mass despite full recovery of vitamin D sufficiency in adulthood (23). Furthermore, a comparison of offspring from reciprocal crosses between strains CC011/Unc (CC011) and CC001/Unc (CC001) showed that this effect differed based on the parental origin of the genome (POG) (23). F_1_ males from CC001-dams x CC011-sires were susceptible to DVD-induced increased adult adiposity, while F_1_ males from CC011-dams x CC001-sires were resistant to DVD-induced increased adult adiposity (23).

Here, we leveraged this unique POG model in an ancillary study to investigate a novel role for vitamin D in the developmental origins of liver metabolic dysfunction. Using liver samples collected from our previously published DVD model (23), we defined DVD-induced changes in liver transcriptional pathways that regulate energy metabolism, cholesterol biosynthesis, inflammation, growth & development, and liver detoxification; examined evidence for a role in perturbing liver metabolic processes; and investigated the role of epigenetic mechanisms in the persistence of these effects. In addition, the use of our Collaborative Cross POG model allowed us to define interindividual differences in susceptibility to DVD-induced adult liver disease.

## 2 Materials and methods

### 2.1 Animal husbandry, dietary treatment, and breeding

All animals were handled in accordance with the Guide for the Care and Use of Laboratory Animals under an approved animal use protocol at the University of North Carolina (UNC) at Chapel Hill. As previously published (23), Collaborative Cross inbred mouse strains, CC001/Unc (CC001) and CC011/Unc (CC011), were purchased from the UNC Systems Genetics Core Facility (Chapel Hill, NC) (24). Inbred CC001 and CC011 virgin dams, aged 4-6 weeks, were placed on one of two isocaloric purified diets for 5 weeks prior to the start of breeding, which is the timeline shown to induce deficiency in mice (25): VDS (vitamin D sufficient diet, 1000 IU/kg vitamin D3, AIN-93G, 110700, Dyets Inc., PA) or VDD (vitamin D depleted diet, 0 IU/kg vitamin D3, AIN-93G, #119266, Dyets Inc., PA). Sires stayed on the VDS diet except when mating with VDD dams (14 days). Dams remained on diet during and after reciprocal mating to CC001 and CC011 sires to generate male F1 offspring that were primarily genetically identical other than different parental origin of the genome (POG) and different mitochondrial and Y chromosomes. (CC001-dam xCC011-sire)F_1_ and (CC011-dam x CC001-sire)F_1_ males were maternally exposed to either VDS or VDD diets from conception to weaning. At PND21, mice were weaned onto standard rodent chow diet (2400 IU/kg vitamin D3; Teklad diet #8604, Harlan Laboratories, Germany) and remained on this vitamin D sufficient diet for 5-6 weeks, which is a timeline shown to be sufficient for vitamin D repletion in mice (25). At 8-9 weeks of age, all F1 adult male offspring were euthanized by CO_2_, and livers were collected and flash-frozen in liquid nitrogen and stored at −80°C. Throughout the study, all mice were housed and maintained at a vivarium temperature of 21-23°C with a 12-h light cycle and ad libitum access to sterilized water and rodent chow (23).

### 2.2 Sample selection (total RNA-sequencing, metabolomics, bisulfite-sequencing)

For RNA-seq, six adult male F_1_ offspring liver samples were selected from at least three different litters for each diet and POG group (n=6/diet/POG, 24 samples total). A subset of these samples (n=3/diet/POG from 3 litters, 12 samples total) were used for the metabolomics and bisulfite-seq experiments. Whole liver samples were pulverized and mixed while frozen and then aliquoted while frozen for each experiment.

### 2.3 Total RNA-Seq

Total RNA was isolated from adult male F1 offspring livers using Trizol reagent following the manufacturer’s protocol (#155960926, Life Technologies, NY). All remaining sample prep and sequencing were performed by the UNC High-Throughput Sequencing Facility (HTSF) at UNC Chapel Hill. RNA was prepped for RNA-Seq using the RNA Clean & concentratorTM-5 kit according to the manufacturer’s protocol (#R1013, Zymo Research, CA). All RNA analytes were assayed for RNA integrity, concentration, and fragment size. Samples for total RNA-Seq were quantified using RNA Qubit (#Q32855, Invitrogen, MA). All samples had a RIN>7.0 (average RIN = 8.9). 500 ng of input RNA was used for library preparation. Fragmentation was performed for 6 minutes at 85°C following the KAPA Stranded RNA-Seq Kit with RiboErase protocol (#8098131702, Illumina Inc., CA). Indexed libraries were prepared and run on NovaSeq XP (#20043131, Illumina Inc., CA) to generate an average of 221 million paired-end reads (100 bp) per sample library. The read length was 2 x 100bp.

Raw sequencing reads were filtered for a quality score > 20 in at least 90% of bases using fastq_quality_filter (version 0.0.14) (26). Sequence adapters were then trimmed using Cutadapt (27) (version 1.12). We then adopted a 2-pass alignment approach utilizing CC001 and CC011 pseudo-references (retrieved from http://www.csbio.unc.edu/CCstatus/index.py?run=Pseudo) to obtain and quantify strain-specific alignments. All samples were first aligned to a hybrid reference genome in which genomic sequences for CC001 and CC011 (based on GRCm37/mm9 coordinates) were concatenated (28), and each chromosome name (chrM) was prepended with the appropriate CC strain. Alignments were performed using STAR (29) (version 2.7.3a), supplying GENCODE vM25 gene annotations as a guide. The splice junction output files were then concatenated, chrM was removed, and then one sample was re-aligned to the initial hybrid reference to insert these newly detected splice junctions and create the final 2-pass hybrid reference genome. All samples were then re-processed onto this 2-pass reference to generate a final set of alignments. Transcript abundance for each strain-gene was then estimated using salmon (30) (version 1.6), and differential expression was detected using DESeq2 (31) (version 1.26.0), where strain and isoform were aggregated by gene symbol.

Differentially expressed genes were identified in two ways using DESeq2 (31) (version 1.26.0): (1) Main diet effects independent of POG - samples from both POGs were combined and data was modeled for diet effects adjusted for POG (~ POG + diet) (**Supplementary Table 1A**); and (2) POG-specific diet effects (**Supplementary Tables 1B, C**) - samples were stratified by POG and modeled for diet effects on each POG separately.

Differentially expressed genes (DEGs) with a nominal p<0.05 were imported into PANTHER (32). DEGs that mapped to the PANTHER mus musculus reference genome (version 2021_03) were assigned to pathways and then tested for pathway overrepresentation using a Fisher’s exact test (PANTHER (32, 33), version 17.0, released 2022-10-13) with FDR<0.1. Gene enrichment analyses were performed on DEGs (nominal p<0.05) using the canonical pathways function in Ingenuity Pathway Analysis (34) (IPA, version 01-20-04 (01-20-04)) with FDR<0.05 (Benjamini and Hochberg method).

Two-way hierarchical clustering of log2 transformed VST-normalized gene expression values was performed in JMP Pro (version 17.0.0 (623769), SAS Institute Inc., Cary, NC, 1989–2021). Gene functions and biological processes were determined using GeneCards (35) (version 5.11.0, build 656) and UniProt (36) (release 2023_01).

### 2.4 Metabolomics

Untargeted profiling of whole liver metabolites was performed using mass spectrometry (Metabolon, Inc (37).) as previously described (38). In brief, four distinct mass spectrometry platforms were utilized in order to capture metabolites across a wide range of varying chemical properties: 1) RP/UPLC-MS/MS with acidic positive ion mode electrospray ionization (ESI) for hydrophilic compounds, 2) RP/UPLC-MS/MS with acidic positive ion mode ESI for hydrophobic compounds, 3) RP/UPLC- MS/MS with basic negative ion mode ESI, and 4) HILIC/UPLC-MS/MS with negative ion mode ESI (38). Metabolites were identified based on their spectrum properties, which included criteria such as m/z and retention time/index. All data underwent extensive quality control by Metabolon, Inc. to account for sample and instrument variability, including interday differences in instrument tuning. To account for these differences while also maintaining appropriate sample variation, raw data were median normalized (38). Samples with measurements that fell below the limit of quantitation were imputed to the limit of quantitation.

Diet and POG effects on metabolite concentrations were analyzed by Principal Component Analysis (PCA) and linear regression modeling. PCA was performed on the normalized and imputed metabolite concentrations using the R package “ropls (ver 1.16.0).” Significant differences in relative concentrations of metabolites were identified in two ways using JMP Pro (version 17.0.0 (623769), SAS Institute Inc., Cary, NC, 1989–2021): (1) Main diet effects independent of POG - samples from both POGs were combined and data was modeled for diet effects adjusted for POG (~ POG + diet); and (2) POG x diet interactive effects were modeled (~ POG + diet + “POG x diet”). Model assumptions for normality (Shapiro-Wilk test) and homoscedasticity (Bartlett’s test) were tested for each model, and metabolites that failed either test (p<0.05) were transformed (log10 or Box-Cox) to best meet assumptions (**Supplementary Tables 1D, E**). In total, after transformation, we performed effect testing on 643 metabolites, including 35 metabolites that did not meet assumptions in the main diet model (1 with significant main diet effect, p<0.05) and 50 metabolites that did not meet assumptions in the interactive model (7 with significant interactive effects, p<0.05). All significant metabolites were reported, but metabolites that did not meet assumptions were not included in the pathway analyses. For pathway analyses, metabolites that met assumptions for linear regression and had significant diet or POG x diet effects (nominal p<0.05) were imported into Metaboanalyst (pathway enrichment function, version 5.0) (39, 40), and pathways with an FDR<0.1 were considered significant. Twenty-five metabolites, predominantly classified as lipids, had no discernable Metaboanalyst-matched name and could not be included in pathway analyses.

### 2.5 Bisulfite-seq

Genomic DNA was isolated using phenol-chloroform extraction as previously described (23). Quantity and quality of DNA were assessed by NanoDrop 2000 spectrometer (Thermo Scientific, DE) and by the Quant-it PicoGreen dsDNA assay (#P7589, Life Technologies, NY). 3 μg dsDNA was used as input, and library preparation was carried out using the SureSelectXT Mouse Methyl-Seq system (Agilent Technologies, CA) following the manufacturer’s protocol. Libraries were multiplexed and sequenced on the Illumina HiSeq2500 (Illumina Inc., CA) at the David H. Murdock Research Institute (DHMRI). Illumina HiSeq single-end 100 bp long reads were generated and trimmed as previously described (41), and the resulting reads were mapped to the UCSC (42) GRCm38/mm10 genome. Only CpGs with ≥10X coverage and a quality score ≥20 were included in the analyses. Single Nucleotide Polymorphisms (SNPs) and other sequences that would potentially interfere with CpG methylation calls were removed from the dataset. ‘Blacklisted’ regions (42) were also removed from the dataset as previously described (41).

MethylKit (version 0.9.5) (43) was used to identify differentially methylated CpGs (DMCs) by logistic regression, as previously described (41). Regression analyses were performed on data after normalizing the coverage between loci using a scaling factor based on the differences in median coverage distributions among CpGs. P-values from the regression analyses were adjusted for multiple comparisons using the SLIM method (FDR<0.05 cutoff) within the MethyKit (43) package. A false discovery rate threshold of 0.05 was applied to identify DMCs. Gene annotations for DMCs (q<0.05) were determined using the UCSC table browser tool (GRCm38/mm10 genome) (44) (**Supplementary Tables 1F, G**).

Differentially methylated genes (DMGs) with a q<0.05 were imported into PANTHER (32). DMGs that mapped to the PANTHER mus musculus reference genome (version 2021_03) were assigned to pathways and then tested for pathway overrepresentation using a Fisher’s exact test (PANTHER (32, 33), version 17.0, released 2022-10-13) with FDR<0.1.

## 3 Results

### 3.1 Developmental vitamin D deficiency (DVD) causes persistent transcriptional dysregulation of liver cholesterol biosynthesis, energy metabolism, growth & development, inflammation, and detoxification pathways

**Figure 1:**
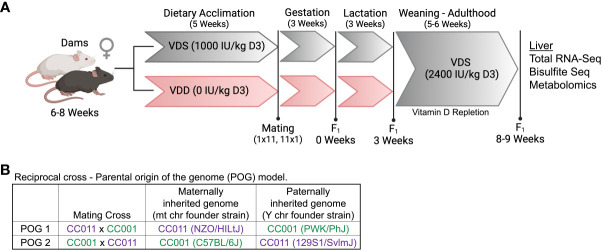
Dietary treatment scheme and POG model. **(A)** Dams from both CC001 and CC011 strains were simultaneously treated with one of two diets (vitamin D sufficient (VDS) or vitamin D depleted (VDD)) for 5 weeks prior to the start of breeding and throughout breeding, gestation, and lactation. All offspring were weaned onto standard rodent chow and remained until adulthood (8-9 weeks of age) when they were euthanized and livers were collected. Figure created with BioRender.com **(B)** A reciprocal cross approach was used to generate F1 offspring (POG1 (CC011xCC001) & POG2 (CC001xCC011)) that differed genetically only for mitochondrial (mt) and sex chromosomes (*csbio.unc.edu*).

To assess the role of DVD in transcriptional programming of molecular pathways of liver disease, we performed an ancillary study on a previously phenotypically characterized DVD cohort of mice (23). Total RNA-seq was performed on livers from 8-week-old adult male offspring (n=6/diet/POG, 24 samples total) treated with either a vitamin D depleted (VDD) or vitamin D sufficient (VDS) diet only from conception until weaning then repleted until adulthood (**Figure 1A**). Adult offspring were assessed for persistent effects of DVD on the liver transcriptome. Bodyweight, fat mass, and vitamin D exposure levels have been previously published for these mice (23). This model incorporates offspring from reciprocal crosses that are genetically identical except for mitochondria and sex chromosomes [POG1-(CC011xCC001)F1 and POG2-(CC001xCC011)F1 males] to characterize interindividual differences related to parental origin of the genome (POG) (**Figure 1B**).

**Figure 2:**
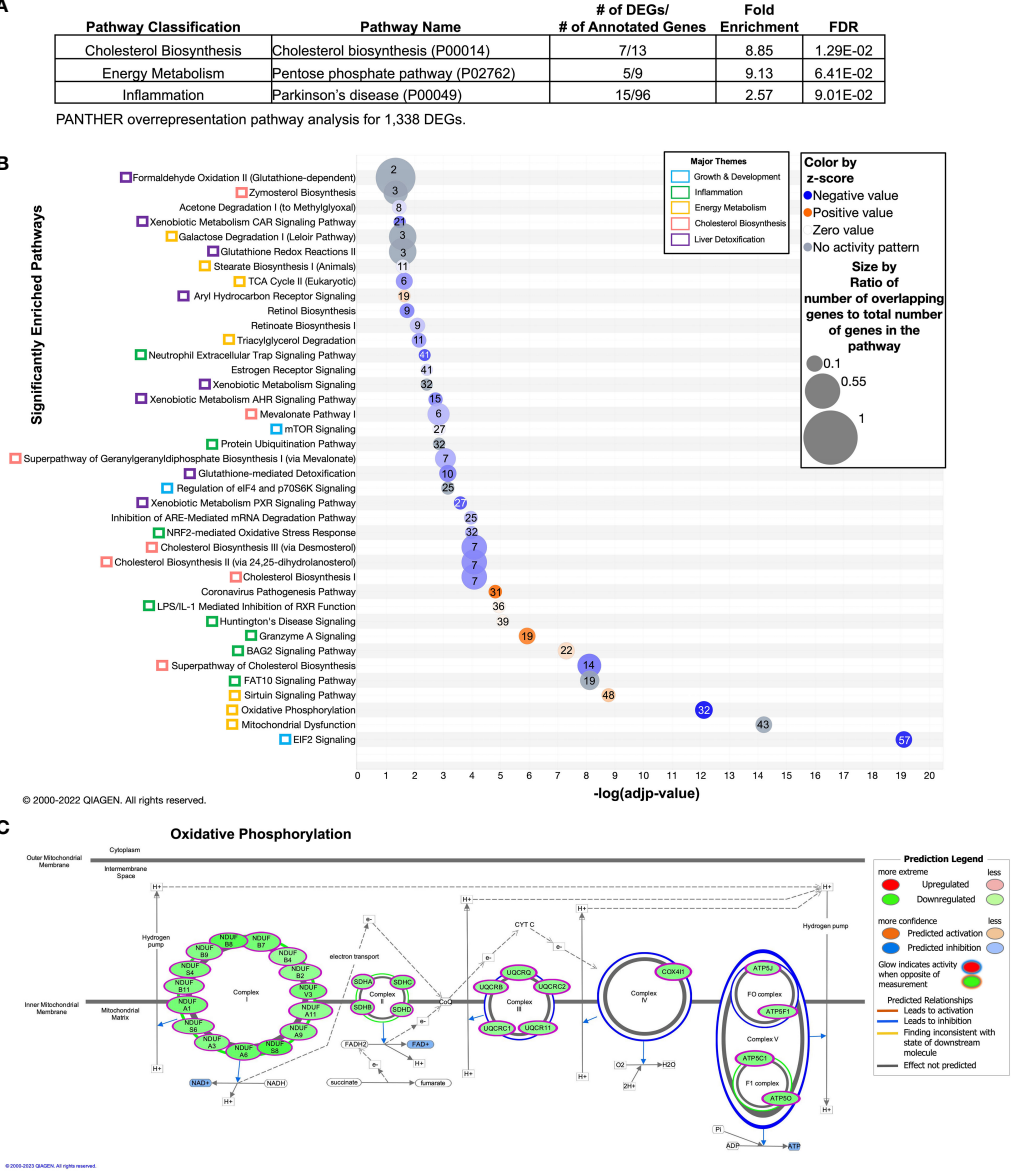
Overrepresented and enriched gene pathways transcriptionally dysregulated by DVD independent of POG. Differentially expressed genes (DEGs) contributing to these pathways are listed in **Supplementary Table 1A**. **(A)** Gene pathway overrepresentation analysis (PANTHER) on 1,338 PANTHER-annotated DEGs with p<0.05 (detected after adjustment for POG). Pathways with FDR<0.1 are shown and classified by biological processes. PANTHER pathway ID numbers are shown in parentheses. **(B, C)** Gene pathway enrichment analysis (IPA) on 1,549 IPA-annotated DEGs with p<0.05 (detected after adjustment for POG). **(B)** Pathways with -log(adjp-value)<0.05 (after Benjamini and Hochberg (BH) correction) are shown and classified by biological process (colored boxes). Pathways are ordered from lowest (bottom) to highest (top) –log(adjp-value). Bubble size indicates the ratio of overlapping genes to the total number of genes within that pathway. Numbers overlaying each pathway bubble represent the number of DVD-altered genes within that pathway. Bubbles are colored by the z-score predictor of pathway activation or inactivation: activated pathways (light to dark orange), unchanged pathways (grey), and deactivated pathways (light to dark blue). **(C)** The 32 DVD-induced DEGs enriched in the oxidative phosphorylation pathway and their functional locations in each of the five electron transport chain complexes. Gene names are shown in green bubbles with the direction of change indicated (green=downregulated; red=upregulated). Data were analyzed through the use of IPA (QIAGEN Inc., https://www.qiagenbioinformatics.com/products/ingenuity-pathway-analysis).

To investigate the effect of DVD independent of POG, we combined POG1 and POG2 datasets and adjusted for POG using linear regression analyses. Of the 53,569 genes queried across the liver genome, 29,471 had detectable expression levels above baseline, and 1,685 were identified as DVD-induced differentially expressed genes (DEGs, nominal p<0.05). Three pathways were significantly overrepresented and downregulated by DVD (PANTHER (32, 33), FDR<0.1): cholesterol biosynthesis (mevalonate pathway), energy metabolism (pentose phosphate pathway - converts glucose to pyruvate), and inflammation (20S proteasome subunits that regulate protein deubiquitination and are triggered by oxidative stress (45)) (**Figure 2A**, **Supplementary Table 2**). Despite relatively small fold changes in gene expression, we observed consistent downregulation of DEGs within these pathways (**Supplementary Table 2**).

We expanded this finding using Ingenuity Pathway Analyses (IPA) to show the enrichment of DEGs in 39 mostly downregulated canonical pathways (FDR<0.05) that regulate cholesterol biosynthesis, energy metabolism, growth & development, inflammation, and liver detoxification (**Figure 2B**). Most notably, this included: (i) predicted downregulation of cholesterol biosynthesis; EIF2 and mTOR signaling, which can inhibit protein synthesis and respond to cellular stress (46); the NRF2-mediated oxidative stress response and subsequent glutathione redox reactions and glutathione-mediated detoxification; and xenobiotic sensing pathways regulated by the nuclear receptors CAR, PXR, and AHR; and (ii) predicted upregulation of the sirtuin signaling pathway, which regulates hepatic lipid and glucose metabolism, stress response, DNA repair mechanisms, and inflammation (47) (**Figure 2B**). The xenobiotic sensing pathways included differentially expressed glutathione-s-transferases (Gsts), which are key phase II liver detoxification enzymes. We also found enrichment of 32 downregulated DEGs in oxidative phosphorylation across all five electron transport chain (ETC) complexes (**Figure 2C**). This finding is consistent with VDR knockdown cell culture models that showed reduced ATP production and oxidative phosphorylation capacity (48–50). Unsupervised hierarchical clustering of the expression profiles for these pathways showed that the differences in gene expression mostly clustered by diet (**Supplementary Figures 1A-H**). However, although both strains exhibited similar responses to DVD on average, the extent of heterogeneity in response differed by strain. For example, across biological replicates, we observed more consistent diet profiles in POG1 for genes regulating oxidative phosphorylation, xenobiotic metabolism, and inflammation (including LPS/IL-1 mediated inflammation, neutrophil trap signaling, granzyme A, and protein ubiquitination). Meanwhile, POG2 had a more consistent diet profile for cholesterol biosynthesis and EIF2 signaling (**Supplementary Figures 1A-H**).

Seven DEGs (including 2 predicted VDR targets, Mkrn2 and Trip13) (51) remained significant after correction for multiple testing (FDR<0.1) and were similarly affected for both POGs (**Supplementary Figure 2A**). These included growth & development and inflammation genes involved in rRNA processing and DNA repair, protein trafficking, and chromosome recombination (downregulated by DVD) (35), and genes involved in protein modification, flagellated motility, and cell cycle regulation and apoptosis (upregulated by DVD) (35) (**Supplementary Figures 2A, B**).

### 3.2 DVD induces unique transcriptional responses on different genomic backgrounds

**Figure 3:**
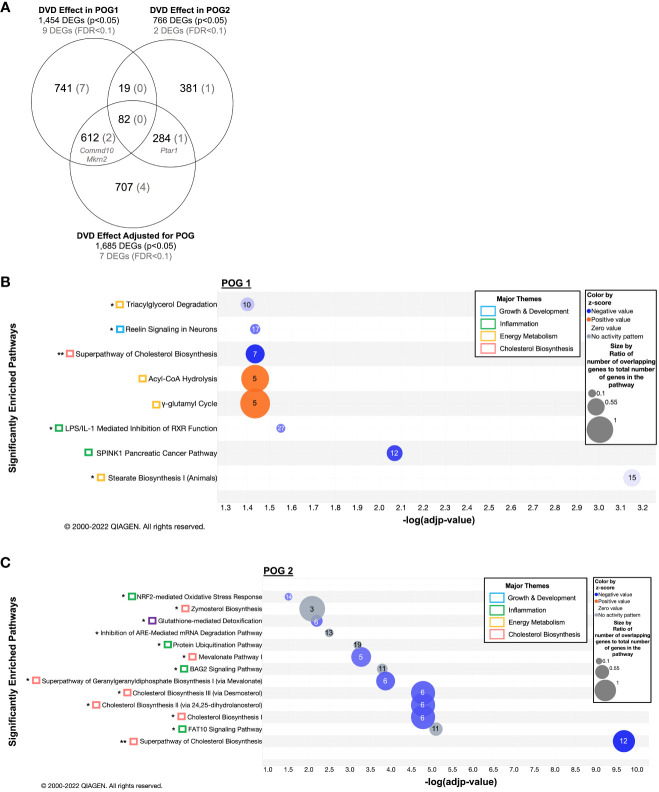
POG-specific enriched gene pathways transcriptionally dysregulated by DVD. **(A)** Venn diagram showing the overlap of DEGs across all three datasets (1) DVD effects in POG1, (2) DVD effects in POG2, and (3) DVD effects for both POGs combined (adjusted for POG). Number of DEGs before (nominal p<0.05) and after (FDR<0.1) correction for multiple testing. **(B)** Gene pathway enrichment analysis (IPA) on 1,360 IPA-annotated DEGs (p<0.05) detected in POG1. **(C)** Gene pathway enrichment analysis (IPA) on 662 IPA annotated DEGs (p<0.05) detected in POG2. **(B, C)** Enriched pathways (-log(adjp-value)<0.05) are shown and classified by biological process (colored boxes). Bubble size indicates the ratio of overlapping genes to the total number of genes within that pathway. Numbers overlaying each pathway bubble represent the number of DVD-altered genes within that pathway. Bubbles are colored by the z-score predictor of pathway activation or inactivation: activated pathways (light to dark orange), unchanged pathways (grey), and deactivated pathways (light to dark blue). Single asterisk (*) indicates pathways also enriched in the DVD independent of POG dataset (**Figure 2**). Double asterisks (**) indicate pathways enriched in all three datasets. DEGs contributing to these pathways are listed in **Supplementary Tables 1B, C**. Data were analyzed through the use of IPA (QIAGEN Inc., https://www.qiagenbioinformatics.com/products/ingenuity-pathway-analysis).

To define the effect of DVD that differed between POG, we analyzed the liver RNA-seq data after stratifying by POG. As expected, the combined POG analyses detected the greatest overall transcriptional response to DVD (1,685 DEGs, **Figure 3A**). However, it failed to find a substantial number of DVD-induced transcriptional effects that were unique to each POG (**Figure 3A**). POG1 mice exhibited 1,454 DEGs (760 new), while POG2 mice had only half as many (766, 400 new) (**Figure 3A**). In addition, each POG exhibited a distinct transcriptional response, with only 13% (101) of the POG2 DEGs overlapping with POG1 (**Figure 3A**).

Despite a greater number of DEGs, POG1 had no significantly overrepresented pathways. POG2 had a significant overrepresentation (FDR<0.1) of the mevalonate pathway of cholesterol biosynthesis, which was previously detected in the combined POG analyses (**Supplementary Table 3**).

DEGs were significantly enriched (FDR<0.1, IPA (34)) in eight pathways for POG1 (**Figure 3B**) and in almost twice as many (13 pathways) for POG2 (**Figure 3C**). Although enrichment and downregulation of cholesterol biosynthesis were common to both POGs, POG2 had a greater number of relevant cholesterol biosynthesis DEGs, including enrichment of zymosterol biosynthesis upstream of cholesterol (**Figure 3C**). POG1 exhibited unique enrichment of downregulated genes involved in pathways that regulate lipid metabolism for energy production (triacylglycerol degradation and stearate biosynthesis), growth & development (reelin signaling), and inflammation (the LPS/IL-1 mediated inhibition of RXR function pathway and a SPINK1 cancer pathway) (**Figure 3B**). POG1 mice also exhibited unique enrichment of upregulated genes involved in acyl-CoA hydrolysis and γ-glutamyl cycle, which are energy metabolism pathways that increase acetyl-CoA and amino acid availability, respectively (**Figure 3B**). In contrast, POG2 mice exhibited unique enrichment of downregulated genes involved in inflammation (NRF2-mediated oxidative stress) and liver detoxification (glutathione-mediated detoxification) (**Figure 3C**).

For POG1, nine DEGs remained significant after correction for multiple testing (FDR<0.1, **Supplementary Figure 3A**): *Mkrn2* and *Commd10* (previously detected), and seven genes that regulate protein modification and metabolism, transcription, membrane trafficking, and inflammation (**Supplementary Figures 3A-C**). For POG2, only two DVD-induced DEGs remained significant after correction for multiple testing (FDR<0.1): *Ptar1* (protein prenylation, previously identified) and *Slc7a15* (amino acid transport) (**Supplementary Figures 4A-C**).

### 3.3 DVD leads to persistent disruption of liver metabolic processes regulating cholesterol biosynthesis and energy metabolism

**Figure 4:**
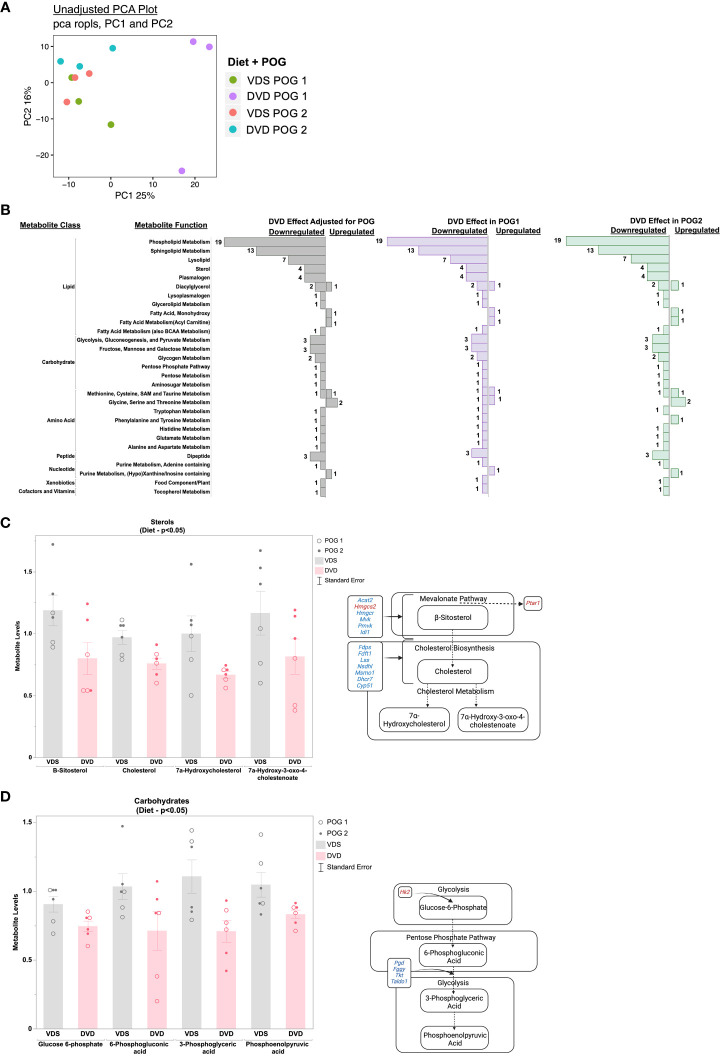
Metabolic pathways dysregulated by DVD independent of POG. **(A)** Principal Component Analysis (PCA) run on all 654 metabolites after normalization and data imputation to the limit of quantitation. Top two principal components (PC1 & PC2) are shown with the percent of variation in metabolite levels they each explain. Each dot in the graph represents an individual mouse liver and colors indicate the diet and POG of that sample. **(B)** Bar graph showing the direction of change for 82 metabolites with significant DVD-induced changes (p<0.05) after adjustment for POG. Bar graphs represent the number of metabolites exhibiting up- or downregulation within each metabolite class and function calculated separately for both POGs combined, for POG1 only, and for POG2 only. Metabolites contributing to these pathways are listed in **Supplementary Table 1D**. **(C)** Metabolite levels for four sterols with known roles in cholesterol biosynthesis are plotted for VDS and DVD groups. POG is indicated by open (POG1) and closed (POG2) circles. Pathway illustration shows the differentially regulated cholesterol metabolites and genes detected within our datasets. Blue indicates downregulation of gene expression by DVD. Red indicates upregulation of gene expression by DVD. **(D)** Metabolite levels for four carbohydrates with known roles in the pentose phosphate pathway are plotted for VDS and DVD groups. POG is indicated by open (POG1) and closed (POG2) circles. Pathway illustration shows the differentially regulated pentose phosphate metabolites and genes detected within our datasets. Blue indicates downregulation of gene expression by DVD. Red indicates upregulation of gene expression by DVD. Illustrations were created with BioRender.com.

We used untargeted metabolomics profiling on livers from a subset of transcriptionally profiled samples (n=3/diet/POG from 3 litters, 12 samples total) to investigate the impact of DVD-induced transcriptional changes on the liver metabolic processes. Principal component analysis (PCA) of the 651 metabolites detected revealed a POG-specific diet effect. PC1 (25%) primarily implicated an effect of DVD on POG1. PC2 (16%), in part, implicated an effect of DVD on POG1 and POG2 (**Figure 4A**). Linear regression analyses to identify the main diet effects (effects of DVD after adjustment for POG) identified 82 metabolites (nominal p<0.05, 0 significant with FDR<0.1) that exhibited mostly reduced abundance on both backgrounds and classified primarily as lipids (~67%), carbohydrates (~16%), and amino acids (~11%) (**Figure 4B**). These metabolites were not significantly enriched for any pathways (Metaboanalyst (39, 40)). However, consistent with the downregulation of cholesterol biosynthesis and pentose phosphate pathway genes in the liver, DVD-treated mice exhibited decreased (nominal p<0.05) levels of liver cholesterol metabolites (**Figure 4C**) and pentose phosphate pathway metabolites (**Figure 4D**).

### 3.4 DVD disrupts distinct metabolic processes on each genomic background

**Figure 5:**
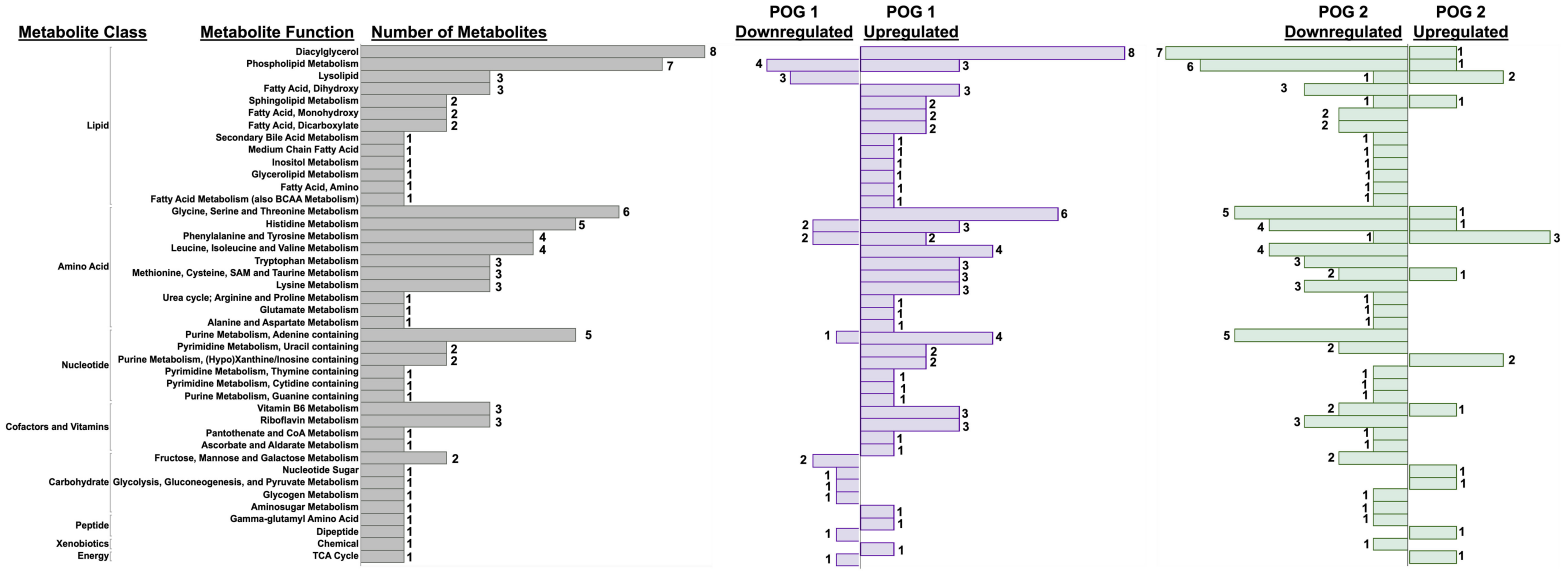
POG-specific effects of DVD on metabolite profiles. Bar graph showing the direction of change for 94 metabolites with significant DVD x POG interactive effects (p<0.05). Bars represent the number of metabolites exhibiting up- or downregulation within each metabolite class and function calculated separately for POG1 and POG2. Metabolites contributing to these pathways are listed in **Supplementary Table 1E**.

**Figure 6:**
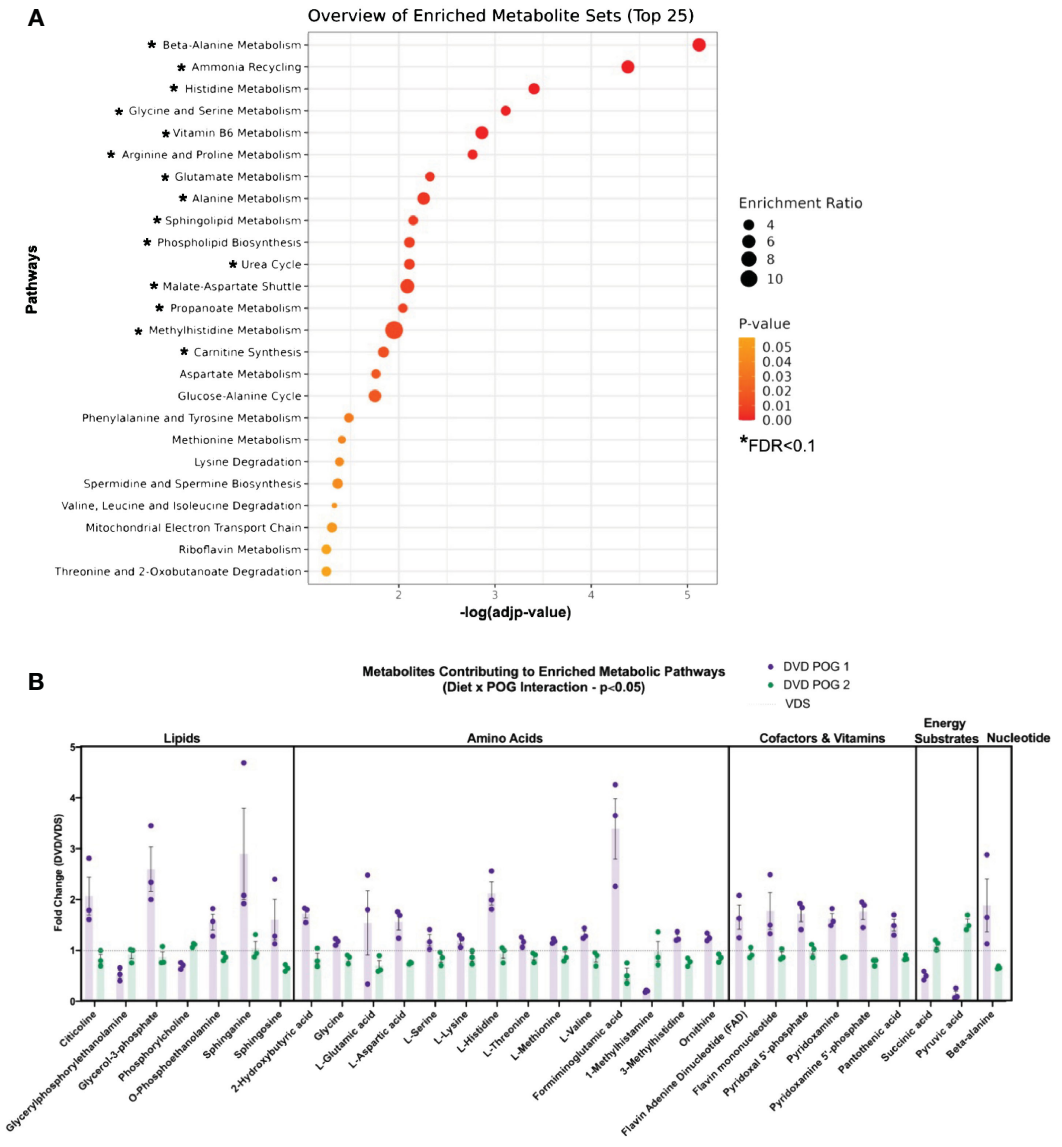
POG-specific enriched metabolite pathways dysregulated by DVD. **(A)** Pathway enrichment analysis (Metaboanalyst) on 71 Metaboanalyst-annotated metabolites with significant DVD x POG interactive effects (p<0.05). Single asterisk (*) indicates pathways with significant enrichment (-log10(FDR)<0.1). Enrichment ratios indicate the ratio of overlapping metabolites over the total number of metabolites within that pathway. **(B)** Fold change (DVD/VDS) of metabolites from Metaboanalyst enrichment analysis. X-axis values >1 mean DVD increased abundance, while values <1 mean DVD decreased abundance.

Based on the POG-specific diet effect observed in the PCA analysis (**Figure 4A**), we used an interactive *diet x POG* linear regression model to identify DVD effects that differed between POGs. We identified 94 metabolites with significant DVD x POG interactive effects (nominal p<0.05, 16 with FDR<0.1 (**Supplementary Table 4**) that were mainly classified as amino acids (~35%), lipids (~33%), and nucleotide intermediates (13%) (**Figure 5**). Strikingly, the two POGs exhibited inverse responses to DVD for most of these metabolites (**Figure 5**). POG1 exhibited increased concentrations of macronutrient metabolites (amino acids, lipids, carbohydrates, etc.) indicative of increased synthesis and increased concentrations of micronutrient cofactors of energy production (e.g., for vitamin B5 & B6), while POG2 concentrations were decreased (**Figure 5**, **Supplementary Figures 5A-E**). This includes effects on branched-chain amino acids ((BCAAs) L-leucine, L-isoleucine, L-valine), energy cofactors (e.g., FAD, flavin mononucleotide, and vitamin B6 metabolites) and substrates (e.g., pyruvic acid, succinic acid, fructose), and purine and pyrimidine metabolites (**Supplementary Figures 5A-E**). Metabolites with DVD x POG interactive effects were enriched (FDR<0.1) in 15 pathways (**Figures 6A, B**, **Supplementary Table 5**). We observed inverse effects on metabolites in these pathways, with increased metabolite abundance for POG1 and decreased abundance for POG2 (**Figure 6B**).

### 3.5 DVD programs distinct liver DNA methylation profiles on each genomic background that explain only a small portion of the transcriptional changes

We previously showed that DVD causes loss of methylation (LOM) in sperm (41). To determine whether the persistent effects of DVD on liver transcription and metabolic processes could be epigenetically programmed by DNA methylation, we used genome-wide bisulfite-sequencing on the metabolically profiled liver samples (n=3/diet/POG from 3 litters, 12 samples total).

**Figure 7:**
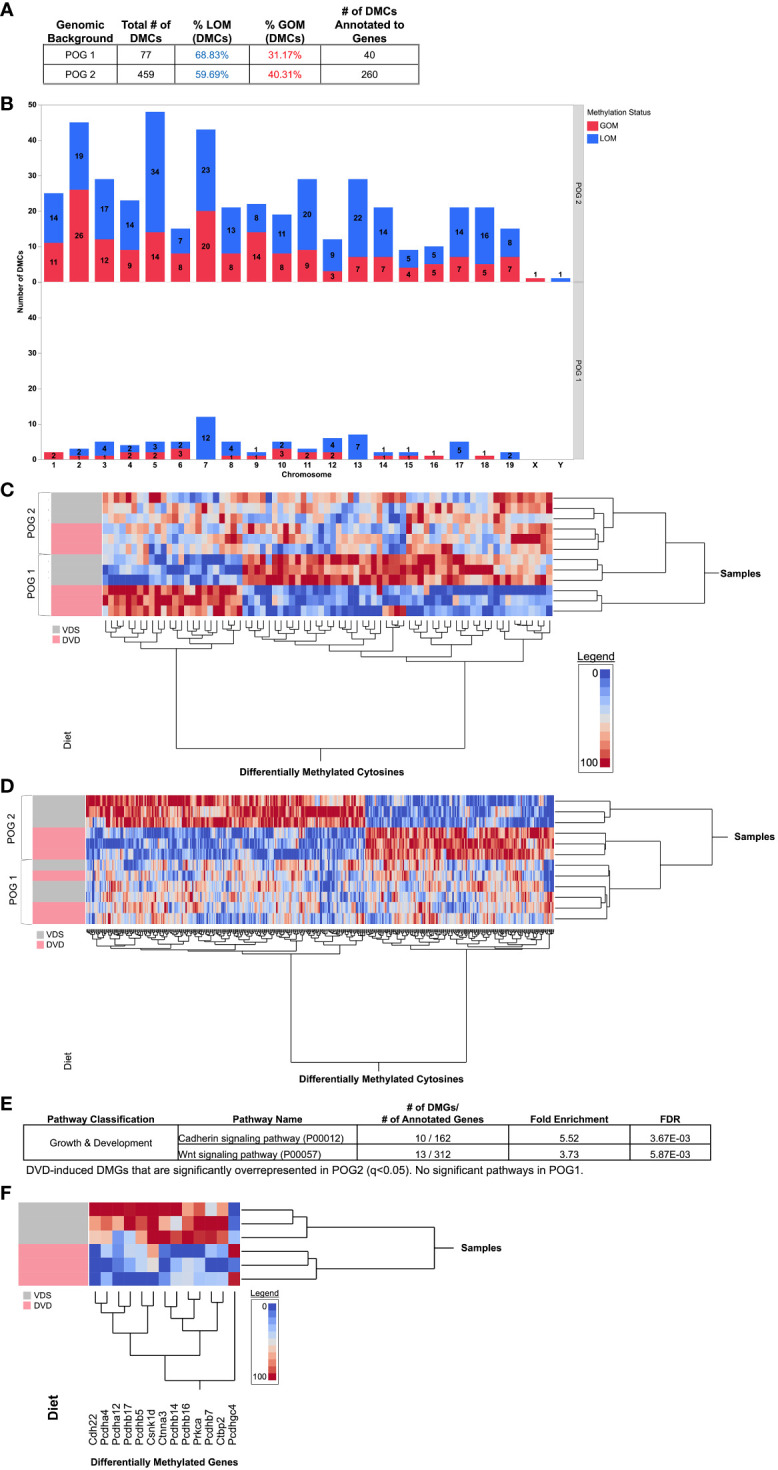
POG-specific differentially methylated cytosines dysregulated by DVD. Blue indicates loss of methylation (LOM). Red indicates gain of methylation (GOM). **(A)** Total numbers of differentially methylated CpGs (DMCs, q<0.05) from genome-wide bisulfite sequencing analysis are shown for POG1 and POG2. **(B)** The distribution of DMCs across chromosomes is shown for POG1 and POG2. **(C, D)** Two-way hierarchical clustering heat maps generated with **(C)** All DMCs detected in POG1 (77, q<0.05) and **(D)** All DMCs detected in POG2 (459, q<0.05). **(E)** Pathway overrepresentation analysis (PANTHER) for POG2. **(F)** Two-way hierarchical clustering heat map for DMGs contributing to significant overrepresentation of Cadherin and Wnt signaling pathways in POG2. DMCs and gene annotations are listed in **Supplementary Tables 1F, G**.

Given the distinct POG response to DVD observed for the transcriptome and metabolome, we identified DVD-induced differentially methylated CpGs (DMCs, q<0.05) for each POG. After removal of CpGs with ≤10X coverage, a quality score ≤20, SNPs, or other sequences that would potentially interfere with methylation calls, 493,989 CpGs were queried across the liver genome. Despite POG1 exhibiting a greater transcriptional response, POG1 exhibited a less robust epigenetic response than POG2 with fewer DMCs (77 DMCs annotated to 40 genes (DMGs)) compared to POG2 (459 DMCs annotated to 260 DMGs) (**Figure 7A**). For both POGs, DMCs were distributed across the genome (**Figure 7B**) and primarily LOM (**Figures 7A, B**). Only one DMC (Chr10:19088363-19088364) and one gene (*Prrc2b*, different DMCs for each POG) were shared by both POGs (**Supplementary Figure 6**). To determine whether the methylation profile induced by DVD was similar between the two POGs (despite poor overlap in “significantly” altered genes), we performed two-way hierarchical clustering of DMCs specific to POG1 (**Figure 7C**) and POG2 (**Figure 7D**). This analysis confirmed that each POG exhibited a distinct DNA methylation profile induced by DVD. Differentially methylated genes (DMGs) mapped to 10 pathways for POG1 (**Supplementary Table 6**) and 59 pathways in POG2 with the top 15 listed in **Supplementary Table 7**. No pathways were overrepresented for POG1. For POG2, we identified significant overrepresentation (FDR<0.1) of growth & development pathways (cadherin signaling pathway and Wnt signaling) (**Figure 7E**, **Supplementary Table 8**) with gene methylation profiles showing strong clustering of LOM by diet (**Figure 7F**).

**Figure 8:**
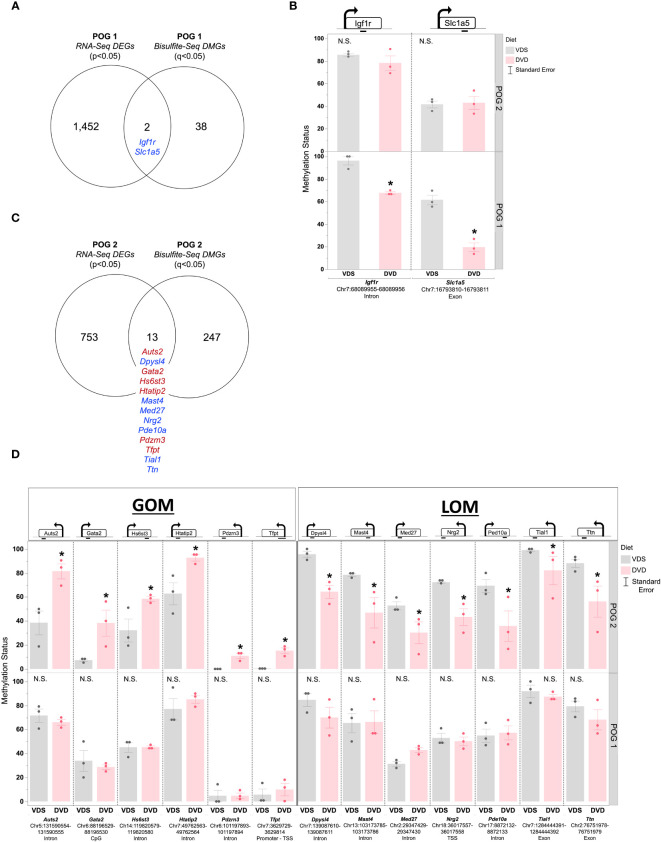
POG-specific overlap between DVD-induced differentially methylated and differentially expressed genes. **(A)** Venn diagram showing the overlap between DEGs (p<0.05) and DMGs (q<0.05) detected in POG1. **(B)** Methylation status is shown for differentially methylated DEGs detected in POG1. **(C)** Venn diagram showing the overlap between DEGs (p<0.05) and DMGs (q<0.05) detected in POG2. **(D)** Methylation status is shown for differentially methylated DEGs detected in POG2. Blue indicates DEG with LOM. Red indicates DEG with GOM. Single asterisk (*) indicates significant differential methylation (q<0.05). N.S. = Not Significant.

There was little overlap between DMGs and DEGs (**Figure 8**, **Supplementary Table 9**). For POG1, only two of the DMGs were also DEGs (**Figure 8A**, **Supplementary Figure 7A**). Both were growth & development genes, *Igf1r* and *Slc1a5*, that exhibited LOM (**Figure 8B**). For POG2, there were 13 growth & development DMGs (including two predicted *Vdr*-targeted genes (*Med27*, *Tfpt*) (52)) with significantly altered liver expression: *Aust2*, *Gata2*, *Hs6st3*, *Htatip2*, *Pdzm3*, *Tfpt* (GOM) and *Dpysl4*, *Mast4*, *Med27*, *Nrg2*, *Ped10a*, *Tial1*, *Ttn* (LOM) (**Figures 8C, D**). All, except *Hs6st3* and *Htatip2*, had increased expression (**Supplementary Figure 7B**).

To determine whether the POG-specific effects of DVD on DNA methylation were driven by differences in regulators of *de novo* methylation (53), we assessed gene expression levels for DNA methyltransferases *Dnmt1*, *Dnmt3a*, and *Dnmt3b*. No significant changes in gene expression were detected for either POG (**Supplementary Figures 8A, B**). Furthermore, no significant changes were detected amongst folate (54) and methionine-related metabolites (51) involved in the establishment and maintenance of DNA methylation (**Supplementary Figure 8C**). Individual SAM and SAH levels were not significantly altered for either POG, however, SAM/SAH ratios were significantly reduced by DVD only in POG1.

## 4 Discussion

**Figure 9:**
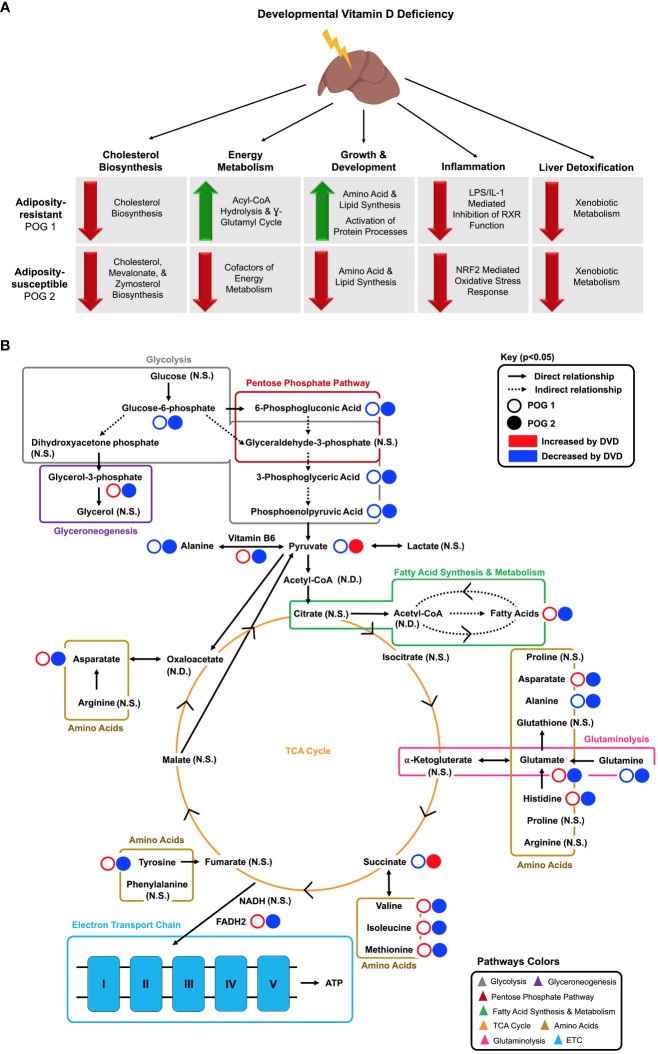
Proposed model for adult liver pathways programmed by early life exposure to DVD. **(A)** Key POG-specific DVD-induced gene expression changes are grouped by major pathway classifications. Red arrows indicate downregulation by DVD. Green arrows indicate upregulation by DVD. Illustration created in part by BioRender.com. **(B)** Key DVD-induced metabolite changes are shown for POG1 (open circles) and POG2 (closed circles) and grouped by metabolic process. Solid arrows indicate a direct relationship between metabolites. Dashed arrows indicate an indirect relationship between metabolites. Red indicates increased by DVD. Blue indicates decreased by DVD. N.S., Not Significant; N.D., Not Detected.

In this study, we demonstrate that developmental vitamin D deficiency programs molecular pathways regulating essential liver functions (**Figure 9A**). This is the first *in vivo* study to define the persistent liver transcriptional profile induced by DVD, to connect these to actively dysregulated liver metabolic processes (metabolome), and to investigate the role of epigenetic mechanisms (DNA methylome) in driving these persistent effects. We found broad transcriptional suppression of gene pathways that are essential to the liver’s ability to respond to environmental stressors, including xenobiotic metabolism, inflammation, and cholesterol biosynthesis. This implicates a potential mechanism by which DVD exposure increases the risk of liver damage and disease. In support of our previous phenotypic findings (23), the Collaborative Cross POG + DVD model demonstrated interindividual differences in liver response to DVD. Although DVD altered similar molecular pathways for both POGs, they differed substantially in penetrance such that the proportion of responders vs. non-responders differed. This was especially striking for the epigenomic and metabolomic responses, where the latter showed inverse effects of DVD on each POG background. This finding shows that the genomic context controls an individual’s response to DVD at the molecular level, demonstrating that a gene x environment mechanism drives the deleterious effects of DVD.

The downregulation of genes controlling cholesterol biosynthesis in the liver was an effect of DVD for both POGs, although penetrance was greater in POG2. Given the essential role of liver-generated cholesterol in cellular structure and function and even vitamin D production, prolonged impaired cholesterol biosynthesis is likely to have deleterious consequences on health. We found subtle but consistent downregulation of genes in the mevalonate pathway of cholesterol biosynthesis and a reduced abundance of liver cholesterol metabolites from this pathway. This effect of DVD is likely a “programmed” effect specific to developmental exposure because we have previously shown that chronic vitamin D deficiency in adult mice from the same strains did not exhibit changes in liver cholesterol levels (38). In humans, vitamin D deficiency has actually been implicated in increased cholesterol (55, 56), but critical longitudinal studies have not been performed to show the time course of deficiency and how cholesterol levels and regulatory pathways respond over time. In fact, prolonged high intracellular cholesterol levels are known to induce mitochondrial dysfunction, leading to inhibition of the mevalonate pathway as a compensatory mechanism for decreasing cholesterol production (57, 58). A similar phenomenon has been demonstrated in VDR KO mice that exhibit decreased serum cholesterol levels at 6 months of age, suggesting that loss of VDR function can also induce attenuation of cholesterol biosynthesis (59). Despite the downregulation of the mevalonate pathway, a downstream target and regulator of protein prenylation, *Ptar1*, was unexplainably upregulated by DVD. *Ptar1* functionality as a subunit of the prenyltransferase, GGTase3, has only recently been recognized and minimally described (60). Therefore, our finding implicates a previously unknown role for *Ptar1* function that is independent of GGTase3 that could explain the upregulation of *Ptar1* expression in the absence of upregulation of the mevalonate pathway. Regardless, our finding of DVD-induced downregulation of the mevalonate pathway has provided a previously undefined role of vitamin D in developmentally programming the molecular mechanisms regulating cholesterol production and, potentially, subsequent protein prenylation.

We found that DVD also primarily downregulated genes controlling the liver’s response to injury and infection. Downregulation of liver detoxification pathways is likely to weaken the liver’s defense system, making it more susceptible to oxidative stress and less effective at deactivating xenobiotic toxicants, all of which would increase susceptibility to liver damage and disease. Here, the penetrance differed by genomic context, with POG1 exhibiting a more consistent liver detoxification and inflammatory response across DVD-treated mice. Both POGs exhibited downregulation of the xenobiotic sensing pathways, CAR, PXR, and AHR, which included five glutathione-s-transferases (*Gst*) (liver detoxification genes); however, this effect was more consistently observed amongst POG1 mice. Similarly, POG1 mice exhibited more consistent downregulation of inflammatory pathways, including neutrophil trap signaling, granzyme A, protein ubiquitination, and LPS/IL-1 mediated inhibition of RXR function. POG2 demonstrated a similar response across granzyme A and protein ubiquitination pathways. Downregulation of these pathways could suggest a suppressed inflammatory response system and subsequent impaired response to injury or infection. In contrast, POG1 mice also exhibited potential pro-inflammatory signatures, including increased expression of *Saa2*, an inflammatory response gene (61, 62); *Mkrn2*, a negative regulator of inflammation (63, 64); and *Spink1*, which has been shown to regulate redox homeostasis (50) and is overexpressed in ~70% of HCC patients (65). Two stress-response pathways that regulate protein translation, EIF2 signaling and mTOR signaling (46), were downregulated by DVD across both POGs, with EIF2 signaling showing the most robust response. Programmed downregulation of EIF2 and mTOR signaling could suggest lower protein translation activity, or it could suggest a suppressed response to cellular stress. However, further data is required to differentiate these scenarios.

DVD altered genes controlling liver development and energy availability. For both POGs, DVD decreased intermediates of glycolysis and the pentose phosphate pathway (**Figure 9B**). However, POG1 demonstrated a unique upregulation of glycerol-3-phosphate, suggesting higher rates of glycerol synthesis via glyceroneogenesis (**Figure 9B**). Other key distinctions between POG1 and POG2 were defined for lipid and amino acid profiles. Lipid synthesis is an energy-demanding processes fueled by lipolysis (66). In POG1, which exhibited a lean phenotype (23), increased levels of long-chain fatty acids (lipids) were detected and supported by decreased levels of energy substrates (carbohydrates & TCA cycle metabolites) (**Figure 9B**). Furthermore, POG1 livers had increased levels of aromatic and BCAAs (**Figure 9B**). These responses were not seen in the adiposity-susceptible POG2 (23), where lipid and amino acid abundance were mainly decreased. One possible explanation for these POG differences in metabolic response could be the upregulation of acyl-CoA hydrolysis and the γ-glutamyl cycle in POG1, which can be upregulated in response to disrupted oxidative phosphorylation as a way to increase cellular availability of acetyl-CoA and amino acid substrates (cysteine, glycine, glutamate), respectively (67). Surprisingly, DVD downregulated oxidative phosphorylation in both POGs, but only POG1 exhibited this compensatory response. Overall, the data indicate that the POG1 response is indicative of increased energy utilization, while the POG2 response indicates decreased energy utilization. Future work is required to determine what characteristic(s) of the parental genome is causing these differences. However, given that most of the changes observed are linked to mitochondrial function, it is likely that genetic differences in the mitochondrial (mt) genes contributed by different founder strains (POG1=NZO/HILtJ; POG2=C57BL/6J) are at least in part responsible.

Our finding that DVD altered the adult liver DNA methylation profile with primarily loss of methylation epimutations supports our previous findings implicating vitamin D in developmental epigenetic programming (23, 41). Strikingly, DVD not only induced a completely different liver DNA methylation profile in each genomic context, but POG2 exhibited substantially greater genomic epigenetic perturbation (~6X as many DMCs and DMGs distributed across the genome). We previously observed a similar pattern of differences in POG epigenetic response at imprinting control regions (ICRs) (23). The overrepresentation of differentially methylated Wnt and Cadherin signaling genes in the liver also mirrors what we found in the germline (sperm) of DVD-treated males from POG2 (41). Surprisingly, the only overlap between DMGs and DEGs was for developmental genes, which suggests this epigenetic response is linked to the developmental response and unlikely to be directly responsible for the majority of transcriptional changes observed. The absence of altered DNA methyltransferase expression in either POG suggests that the methylation changes induced by DVD and the POG-specific differences in DVD response are not due to current transcriptional suppression of the primary DNA methyltransferase genes. This does not rule out differences at earlier developmental time points, which were not measured here. Lastly, while individual SAM and SAH metabolites were not significantly altered in either POG, SAM/SAH ratios were significantly reduced in POG1, which could suggest a lower methylation capacity (68).

In conclusion, our data show that early life exposure to vitamin D deficiency plays a novel role in programming the molecular underpinnings of adult liver function and health. These findings specifically support the role of vitamin D in the developmental origins of adult liver health and diseases such as NAFLD and HCC. The widespread DVD-induced downregulation of pathways controlling essential liver processes suggests overarching suppression of liver function. However, the small effect sizes of most of the transcriptional changes imply that DVD alone may not cause disease but likely impairs the ability of the liver to utilize these essential protective pathways in response to other stressors. Our findings of distinct responses to DVD based on POG mirror our earlier findings (23, 41) and further drive the need to understand how underlying mitochondrial differences could contribute to interindividual differences in liver disease. Importantly, future studies are needed to define the pathological effects of molecular changes driven by DVD.

## Data availability statement

The data presented in the study are deposited in the Sequence Read Archive (SRA) repository, accession number PRJNA1104589; and the Mendeley Data Repository, doi: 10.17632/gt338g42b3.1.

## Ethics statement

The animal study was approved by The University of North Carolina at Chapel Hill Institutional Animal Care and Use Committee. The study was conducted in accordance with the local legislation and institutional requirements.

## Author contributions

MK: Writing – review & editing, Validation, Writing – original draft, Visualization, Investigation, Formal analysis. JX: Data curation, Writing – review & editing, Software, Methodology, Investigation, Formal analysis, Conceptualization. ME: Methodology, Writing – review & editing. RG: Methodology, Writing – review & editing. SAS: Investigation, Writing – review & editing. SM: Methodology, Writing – review & editing. CB: Methodology, Writing – review & editing. SJS: Methodology, Writing – review & editing. LT: Resources, Writing – review & editing, Methodology. WV: Resources, Writing – review & editing, Methodology. RR: Methodology, Writing – review & editing, Funding acquisition. JMS: Validation, Supervision, Resources, Data curation, Writing – review & editing, Software, Methodology, Formal analysis. FI: Writing – review & editing, Investigation, Writing – original draft, Supervision, Resources, Project administration, Methodology, Funding acquisition, Formal analysis, Data curation, Conceptualization.

## References

[1] Lu Q Tian X Wu H Huang J Li M Mei Z. Metabolic changes of hepatocytes in NAFLD. Front Physiol. (2021) 12:710420. doi: 10.3389/fphys.2021.710420 34526911 PMC8437340

[2] El-Kader SMA. Non-alcoholic fatty liver disease: The diagnosis and management. World J Hepatol. (2015) 7:846. doi: 10.4254/wjh.v7.i6.846 25937862 PMC4411527

[3] Luci C Bourinet M Leclère PS Anty R Gual P. Chronic inflammation in non-alcoholic steatohepatitis: molecular mechanisms and therapeutic strategies. Front Endocrinol. (2020) 11:597648. doi: 10.3389/fendo.2020.597648 PMC7771356 33384662

[4] Gofton C Upendran Y Zheng MH George J. MAFLD: How is it different from NAFLD? Clin Mol Hepatol. (2023) 29:S17–31. doi: 10.3350/cmh.2022.0367 PMC10029949 36443926

[5] Cotter TG Rinella M. Nonalcoholic fatty liver disease 2020: the state of the disease. Gastroenterology. (2020) 158:1851–64. doi: 10.1053/j.gastro.2020.01.052 32061595

[6] Dhamija E Paul S Kedia S. Non-alcoholic fatty liver disease associated with hepatocellular carcinoma: An increasing concern. Indian J Med Res. (2019) 149:9. doi: 10.4103/ijmr.IJMR_1456_17 31115369 PMC6507546

[7] Geh D Anstee QM Reeves HL. NAFLD-associated HCC: progress and opportunities. J Hepatocell Carcinoma. (2021) 8:223–39. doi: 10.2147/JHC.S272213 PMC8041650 33854987

[8] Polyzos SA Mantzoros CS. Making progress in nonalcoholic fatty liver disease (NAFLD) as we are transitioning from the era of NAFLD to dys-metabolism associated fatty liver disease (DAFLD). Metabolism. (2020) 111:154318. doi: 10.1016/j.metabol.2020.154318 PMC7372254 32707055

[9] De Boo HA Harding JE. The developmental origins of adult disease (Barker) hypothesis. Aust N Z J Obstet Gynaecol. (2006) 46:4–14. doi: 10.1111/j.1479-828X.2006.00506.x 16441686

[10] Itoh H Kanayama N. Developmental Origins of Nonalcoholic Fatty Liver Disease (NAFLD). In: Kubota T Fukuoka H, editors. Developmental Origins of Health and Disease (DOHaD). Springer Singapore, Singapore (2018). p. 29–39. doi: 10.1007/978-981-10-5526-3_4 29956192

[11] Wesolowski SR Kasmi KCE Jonscher KR Friedman JE. Developmental origins of NAFLD: a womb with a clue. Nat Rev Gastroenterol Hepatol. (2017) 14:81–96. doi: 10.1038/nrgastro.2016.160 27780972 PMC5725959

[12] Sharma SS Jangale NM Harsulkar AM Gokhale MK Joshi BN. Chronic maternal calcium and 25-hydroxyvitamin D deficiency in Wistar rats programs abnormal hepatic gene expression leading to hepatic steatosis in female offspring. J Nutr Biochem. (2017) 43:36–46. doi: 10.1016/j.jnutbio.2017.01.008 28219837

[13] Zhang H Chu X Huang Y Li G Wang Y Li Y. Maternal vitamin D deficiency during pregnancy results in insulin resistance in rat offspring, which is associated with inflammation and Iκbα methylation. Diabetologia (2014) 57(10):2165–72. doi: 10.1007/s00125-014-3316-7 24985146

[14] Newton KP Feldman HS Chambers CD Wilson L Behling C Clark JM. Low and high birth weights are risk factors for nonalcoholic fatty liver disease in children. J Pediatr. (2017) 187:141–146.e1. doi: 10.1016/j.jpeds.2017.03.007 28366357 PMC5533631

[15] Faienza MF Brunetti G Ventura A D’Aniello M Pepe T Giordano P. Nonalcoholic fatty liver disease in prepubertal children born small for gestational age: influence of rapid weight catch-up growth. Horm Res Paediatr. (2013) 79:103–9. doi: 10.1159/000347217 23466642

[16] Zheng J Liu X Zheng B Zheng Z Zhang H Zheng J. Maternal 25-hydroxyvitamin D deficiency promoted metabolic syndrome and downregulated nrf2/CBR1 pathway in offspring. Front Pharmacol. (2020) 11:97. doi: 10.3389/fphar.2020.00097 32184720 PMC7058637

[17] Lundy K Greally JF Essilfie-Bondzie G Olivier JB Doña-Termine R Greally JM. Vitamin D deficiency during development permanently alters liver cell composition and function. Front Endocrinol. (2022) 13:860286. doi: 10.3389/fendo.2022.860286 PMC9133936 35634491

[18] Yates N Crew RC Wyrwoll CS. Vitamin D deficiency and impaired placental function: potential regulation by glucocorticoids? Reproduction. (2017) 153:R163–71. doi: 10.1530/REP-16-0647 28137896

[19] Khalessi N Kalani M Araghi M Farahani Z. The relationship between maternal vitamin D deficiency and low birth weight neonates. J Family Reprod Health. (2015) 9(3):113–7. PMC4662754 26622309

[20] Hu Z Tang L Xu HL. Maternal vitamin D deficiency and the risk of small for gestational age: A meta-analysis. Iran J Public Health (2018) 47(12):1785–95. PMC6379614 30788292

[21] Mericq V Martinez-Aguayo A Uauy R Iñiguez G van der Steen M Hokken-Koelega A. Long-term metabolic risk among children born premature or small for gestational age. Nat Rev Endocrinol. (2017) 13:50–62. doi: 10.1038/nrendo.2016.127 27539244

[22] Möllers LS Yousuf EI Hamatschek C Morrison KM Hermanussen M Fusch C. Metabolic-endocrine disruption due to preterm birth impacts growth, body composition, and neonatal outcome. Pediatr Res. (2022) 91:1350–60. doi: 10.1038/s41390-021-01566-8 PMC9197767 34040160

[23] Xue J Schoenrock SA Valdar W Tarantino LM Ideraabdullah FY. Maternal vitamin D depletion alters DNA methylation at imprinted loci in multiple generations. Clin Epigenet. (2016) 8:107. doi: 10.1186/s13148-016-0276-4 PMC5062906 27777636

[24] Welsh CE Miller DR Manly KF Wang J McMillan L Morahan G. Status and access to the Collaborative Cross population. Mamm Genome. (2012) 23:706–12. doi: 10.1007/s00335-012-9410-6 PMC3463789 22847377

[25] Belenchia AM Johnson SA Kieschnick AC Rosenfeld CS Peterson CA. Time course of vitamin D depletion and repletion in reproductive-age female C57BL/6 mice. Comp Med. (2017) 67:8. PMC5713162 29212579

[26] Hannon GJ. FASTX-Toolkit (2010). Available online at: http://hannonlab.cshl.edu/fastx_toolkit/.

[27] Martin M. Cutadapt removes adapter sequences from high-throughput sequencing reads. EMBnet.journal. (2011) 17:10. doi: 10.14806/ej.17.1

[28] Keele GR Quach BC Israel JW Chappell GA Lewis L Safi A. Integrative QTL analysis of gene expression and chromatin accessibility identifies multi-tissue patterns of genetic regulation. PloS Genet. (2020) 16:e1008537. doi: 10.1371/journal.pgen.1008537 31961859 PMC7010298

[29] Dobin A Davis CA Schlesinger F Drenkow J Zaleski C Jha S. STAR: ultrafast universal RNA-seq aligner. Bioinforma Oxf Engl. (2013) 29:15–21. doi: 10.1093/bioinformatics/bts635 PMC3530905 23104886

[30] Patro R Duggal G Love MI Irizarry RA Kingsford C. Salmon provides fast and bias-aware quantification of transcript expression. Nat Methods. (2017) 14:417–9. doi: 10.1038/nmeth.4197 PMC5600148 28263959

[31] Love MI Huber W Anders S. Moderated estimation of fold change and dispersion for RNA-seq data with DESeq2. Genome Biol. (2014) 15:550. doi: 10.1186/s13059-014-0550-8 25516281 PMC4302049

[32] Thomas PD Ebert D Muruganujan A Mushayahama T Albou L Mi H. PANTHER : Making genome-scale phylogenetics accessible to all. Protein Sci. (2022) 31:8–22. doi: 10.1002/pro.4218 34717010 PMC8740835

[33] Mi H Muruganujan A Huang X Ebert D Mills C Guo X. Protocol Update for large-scale genome and gene function analysis with the PANTHER classification system (v.14.0). Nat Protoc. (2019) 14:703–21. doi: 10.1038/s41596-019-0128-8 PMC6519457 30804569

[34] Krämer A Green J Pollard J Tugendreich S. Causal analysis approaches in Ingenuity Pathway Analysis. Bioinformatics. (2014) 30:523–30. doi: 10.1093/bioinformatics/btt703 PMC3928520 24336805

[35] Safran M Rosen N Twik M BarShir R Stein TI Dahary D. The GeneCards Suite. In: Abugessaisa I Kasukawa T, editors. Practical Guide to Life Science Databases. Springer Nature Singapore, Singapore (2021). p. 27–56. doi: 10.1007/978-981-16-5812-9_2

[36] The UniProt Consortium Bateman A Martin MJ Orchard S Magrane M Ahmad S. UniProt: the universal protein knowledgebase in 2023. Nucleic Acids Res. (2023) 6;51:D523–31. doi: 10.1093/nar/gkac1052 PMC9825514 36408920

[37] Evans AM Bridgewater BR Liu Q Mitchell MW Robinson RJ Dai H. High resolution mass spectrometry improves data quantity and quality as compared to unit mass resolution mass spectrometry in high-throughput profiling metabolomics. J Postgenomics Drug biomark Dev. (2014) 4:2. doi: 10.4172/2153-0769

[38] Xue J Hutchins EK Elnagheeb M Li Y Valdar W McRitchie S. Maternal liver metabolic response to chronic vitamin D deficiency is determined by mouse strain genetic background. Curr Dev Nutr. (2020) 4(8):nzaa106. doi: 10.1093/cdn/nzaa106 32851199 PMC7439094

[39] Xia J Psychogios N Young N Wishart DS. MetaboAnalyst: a web server for metabolomic data analysis and interpretation. Nucleic Acids Res. (2009) 37:W652–60. doi: 10.1093/nar/gkp356 PMC2703878 19429898

[40] Xia J Psychogios N Young N Wishart DS. MetaboAnalyst: a web server for metabolomic data analysis and interpretation. Nucleic Acids Res. (2009) 37(Web Server issue):W652–60. doi: 10.1093/nar/gkp356 PMC2703878 19429898

[41] Xue J Gharaibeh RZ Pietryk EW Brouwer C Tarantino LM Valdar W. Impact of vitamin D depletion during development on mouse sperm DNA methylation. Epigenetics. (2018) 13:959–74. doi: 10.1080/15592294.2018.1526027 PMC6284778 30239288

[42] The ENCODE Project Consortium. An integrated encyclopedia of DNA elements in the human genome. Nature. (2012) 489:57–74. doi: 10.1038/nature11247 22955616 PMC3439153

[43] Akalin A Kormaksson M Li S Garrett-Bakelman FE Figueroa ME Melnick A. methylKit: a comprehensive R package for the analysis of genome-wide DNA methylation profiles. Genome Biol. (2012) 13:R87. doi: 10.1186/gb-2012-13-10-r87 23034086 PMC3491415

[44] Karolchik D. The UCSC Browser data retrieval tool. Nucleic Acids Res. (2004) 32:493D – 496. doi: 10.1093/nar/gkh103 PMC308837 14681465

[45] Pickering AM Davies KJ. Degradation of damaged proteins: the main function of the 20S proteasome. Prog Mol Biol Transl Sci. (2012) 109:227–48. doi: 10.1016/B978-0-12-397863-9.00006-7 PMC3710712 22727423

[46] Ghosh A Shcherbik N. Effects of oxidative stress on protein translation: implications for cardiovascular diseases. Int J Mol Sci. (2020) 21:2661. doi: 10.3390/ijms21082661 32290431 PMC7215667

[47] Kemper JK Choi SE Kim DH. Sirtuin 1 deacetylase: a key regulator of hepatic lipid metabolism. Vitam Horm. (2013) 91:385–404. doi: 10.1016/B978-0-12-407766-9.00016-X PMC4976444 23374725

[48] Latham CM Brightwell CR Keeble AR Munson BD Thomas NT Zagzoog AM. Vitamin D promotes skeletal muscle regeneration and mitochondrial health. Front Physiol. (2021) 12:11. doi: 10.3389/fphys.2021.660498 PMC8079814 33935807

[49] Ashcroft SP Bass JJ Kazi AA Atherton PJ Philp A. The vitamin D receptor (VDR) regulates mitochondrial function in C2C12 myoblasts. Am J Physiol Cell Physiol. (2020) 318(3):C536–41. doi: 10.1101/872127 PMC7099523 31940245

[50] Bass JJ Kazi AA Deane CS Nakhuda A Ashcroft SP Brook MS. The mechanisms of skeletal muscle atrophy in response to transient knockdown of the vitamin D receptor in vivo. J Physiol. (2021) 599:963–79. doi: 10.1113/JP280652 PMC7986223 33258480

[51] Lauinger L Kaiser P. Sensing and signaling of methionine metabolism. Metabolites. (2021) 11:83. doi: 10.3390/metabo11020083 33572567 PMC7912243

[52] Rouillard AD Gundersen GW Fernandez NF Wang Z Monteiro CD McDermott MG. The harmonizome: a collection of processed datasets gathered to serve and mine knowledge about genes and proteins. Database. (2016) 2016:baw100. doi: 10.1093/database/baw100 27374120 PMC4930834

[53] Leppert S Matarazzo M. *De novo* DNMTs and DNA methylation: novel insights into disease pathogenesis and therapy from epigenomics. Curr Pharm Des. (2014) 20:1812–8. doi: 10.2174/13816128113199990534 23888951

[54] Crider KS Yang TP Berry RJ Bailey LB. Folate and DNA methylation: A review of molecular mechanisms and the evidence for folate’s role. Adv Nutr. (2012) 3:21–38. doi: 10.3945/an.111.000992 22332098 PMC3262611

[55] Kim MR Jeong SJ. Relationship between vitamin D level and lipid profile in non-obese children. Metabolites. (2019) 9:125. doi: 10.3390/metabo9070125 31262034 PMC6680594

[56] Dibaba DT. Effect of vitamin D supplementation on serum lipid profiles: a systematic review and meta-analysis. Nutr Rev. (2019) 77:890–902. doi: 10.1093/nutrit/nuz037 31407792

[57] Wall CTJ Lefebvre G Metairon S Descombes P Wiederkehr A Santo-Domingo J. Mitochondrial respiratory chain dysfunction alters ER sterol sensing and mevalonate pathway activity. J Biol Chem. (2022) 298:101652. doi: 10.1016/j.jbc.2022.101652 35101444 PMC8892029

[58] Pisanti S Rimondi E Pozza E Melloni E Zauli E Bifulco M. Prenylation defects and oxidative stress trigger the main consequences of neuroinflammation linked to mevalonate pathway deregulation. Int J Environ Res Public Health. (2022) 19:9061. doi: 10.3390/ijerph19159061 35897423 PMC9332440

[59] Weber K Erben RG. Differences in triglyceride and cholesterol metabolism and resistance to obesity in male and female vitamin D receptor knockout mice: Lipid metabolism and obesity in VDR knockout mice. J Anim Physiol Anim Nutr. (2013) 97:675–83. doi: 10.1111/j.1439-0396.2012.01308.x 22548652

[60] Kuchay S Wang H Marzio A Jain K Homer H Fehrenbacher N. GGTase3 is a newly identified geranylgeranyltransferase targeting a ubiquitin ligase. Nat Struct Mol Biol. (2019) 26:628–36. doi: 10.1038/s41594-019-0249-3 PMC6609460 31209342

[61] Wu JL Su TH Chen PJ Chen YR. Acute-phase serum amyloid A for early detection of hepatocellular carcinoma in cirrhotic patients with low AFP level. Sci Rep. (2022) 12:5799. doi: 10.1038/s41598-022-09713-9 35388082 PMC8986837

[62] De Buck M Gouwy M Wang JM Van Snick J Proost P Struyf S. The cytokine-serum amyloid A-chemokine network. Cytokine Growth Factor Rev. (2016) 30:55–69. doi: 10.1016/j.cytogfr.2015.12.010 26794452 PMC7512008

[63] Shin C Ito Y Ichikawa S Tokunaga M Sakata-Sogawa K Tanaka T. MKRN2 is a novel ubiquitin E3 ligase for the p65 subunit of NF-κB and negatively regulates inflammatory responses. Sci Rep. (2017) 7:46097. doi: 10.1038/srep46097 28378844 PMC5380948

[64] Zhang Y Cui N Zheng G. Ubiquitination of P53 by E3 ligase MKRN2 promotes melanoma cell proliferation. Oncol Lett. (2020) 19(3):1975–84. doi: 10.3892/ol.2020.11261 PMC7039176 32194692

[65] Lin TC. Functional roles of SPINK1 in cancers. Int J Mol Sci. (2021) 22:3814. doi: 10.3390/ijms22083814 33916984 PMC8067593

[66] Lass A Zimmermann R Oberer M Zechner R. Lipolysis – A highly regulated multi-enzyme complex mediates the catabolism of cellular fat stores. Prog Lipid Res. (2011) 50:14–27. doi: 10.1016/j.plipres.2010.10.004 21087632 PMC3031774

[67] Sánchez-González C Nuevo-Tapioles C Herrero Martín JC Pereira MP Serrano Sanz S Ramírez De Molina A. Dysfunctional oxidative phosphorylation shunts branched-chain amino acid catabolism onto lipogenesis in skeletal muscle. EMBO J. (2020) 39:e103812. doi: 10.15252/embj.2019103812 32488939 PMC7360968

[68] Bravo AC Aguilera MNL Marziali NR Moritz L Wingert V Klotz K. Analysis of S-adenosylmethionine and S-adenosylhomocysteine: method optimisation and profiling in healthy adults upon short-term dietary intervention. Metabolites. (2022) 12:373. doi: 10.3390/metabo12050373 35629877 PMC9143066

