# Supplementary material for: Early life exposure to vitamin D deficiency impairs molecular mechanisms that regulate liver cholesterol biosynthesis, energy metabolism, inflammation, and detoxification
Source: Front Endocrinol (Lausanne). 2024 May 10;15:1335855. doi: 10.3389/fendo.2024.1335855 (PMC11116800; doi:10.3389/fendo.2024.1335855)
Supplement: Supplementary file 1 [file DataSheet_1.pdf]

**Supplemental Figure 1. Two-way hierarchical clustering of pathways with enrichment of genes by DVD.**

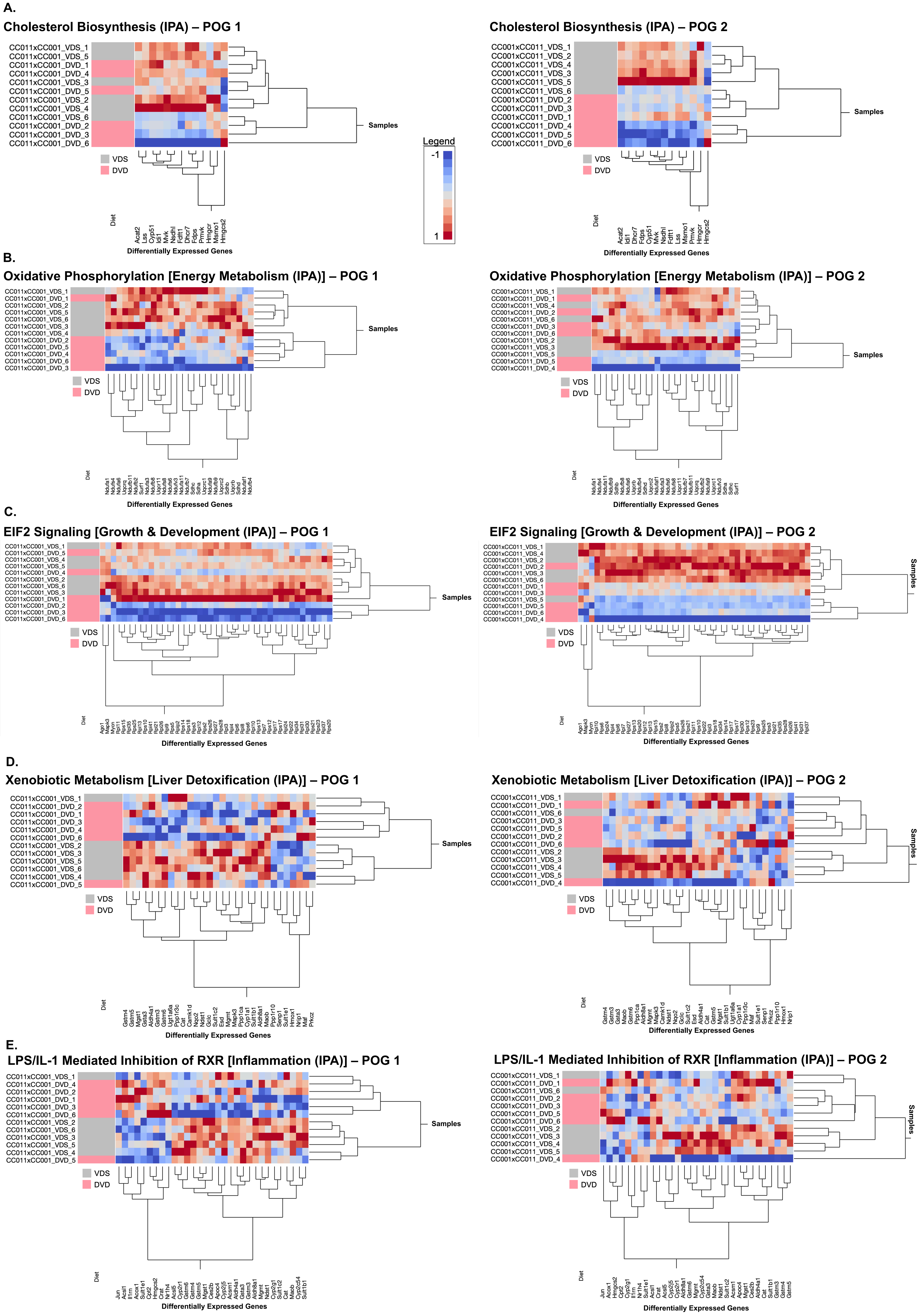

**Supplemental Figure 1. Two-way hierarchical clustering of pathways with enrichment of genes by DVD.**

**F.**

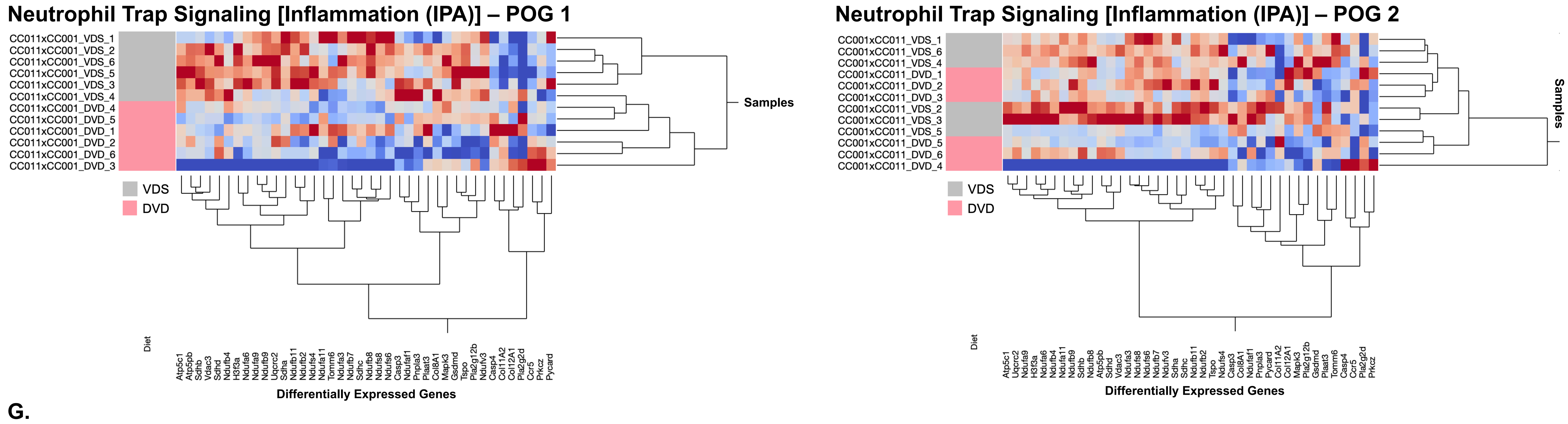


**G.**

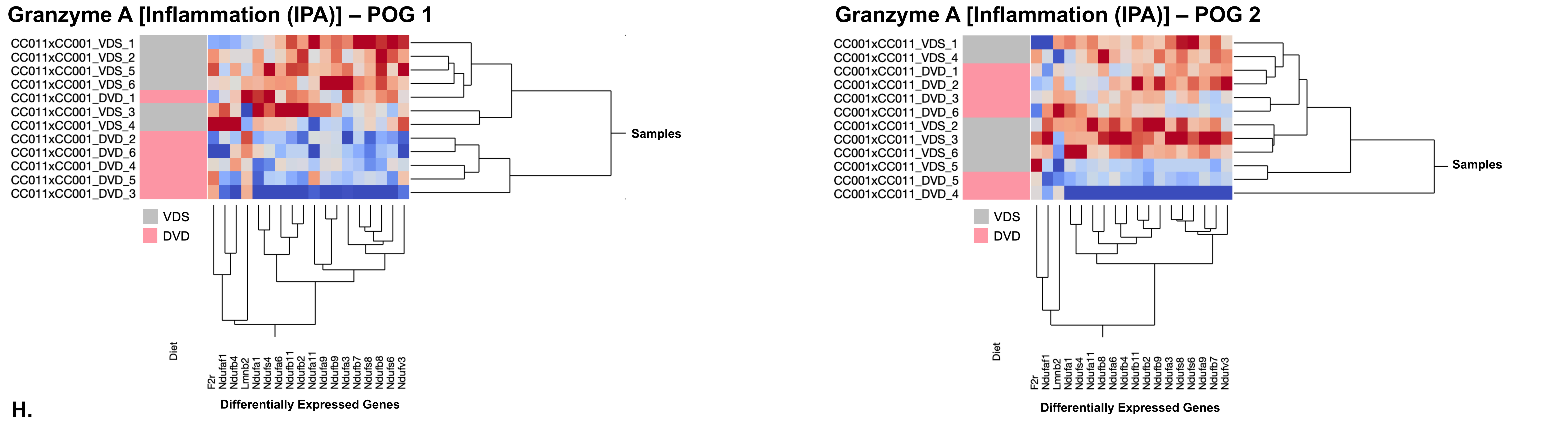


**H.**

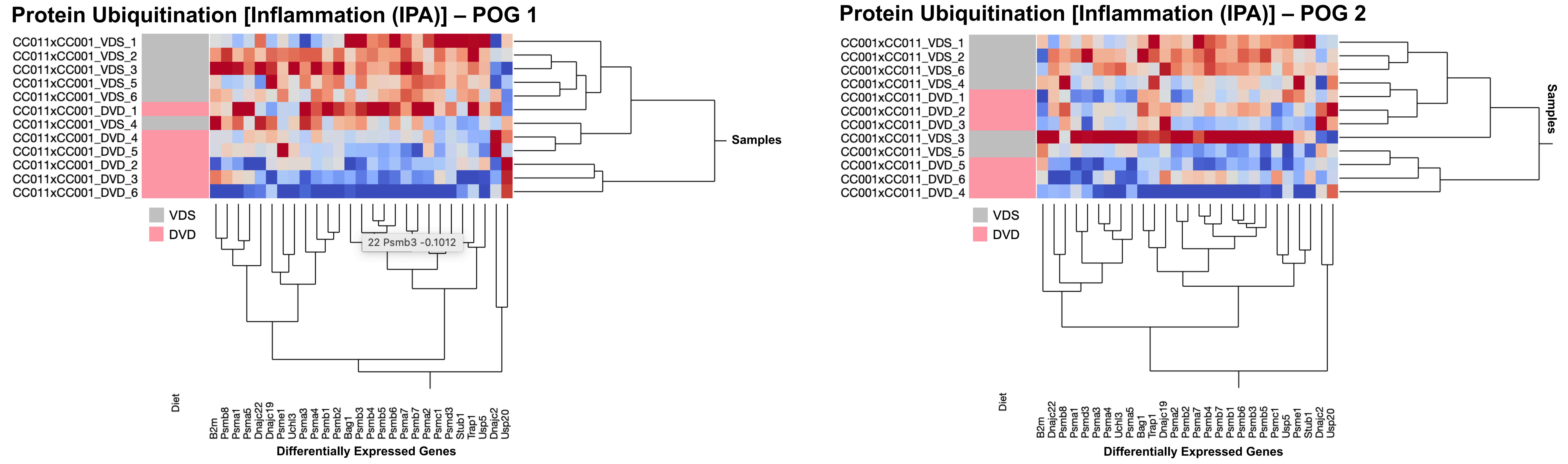


**Supplemental Figure 1. Two-way hierarchical clustering heat maps of DEGs (p<0.05) enriched for pathways altered by DVD**. POG1 (left) and POG2 (right). **(A)** Cholesterol biosynthesis. **(B)** Oxidative phosphorylation. **(C)** EIF2 signaling. **(D)** Xenobiotic metabolism. **(E)** LPS/IL-1 mediated inhibition of RXR function. **(F)** Neutrophil trap signaling. **(G)** Granzyme A signaling. **(H)** Protein ubiquitination. Blue indicates downregulation by DVD. Red indicates upregulation by DVD.

**Supplemental Figure 2. Diet Effects Independent of POG.**

A.

| Gene Pathway Classification | Gene Name | Log2 Fold Change (DVD/VDS) | FDR | Gene Function (GeneCards (RRID:SCR_002773)) | Major Biological Process |
|---|---|---|---|---|---|
| Growth & Development | *Commd10* | -0.38 | 3.00E-03 | *COMM Domain Containing 10* – regulates transcription and protein trafficking | Protein trafficking |
| | *Ptar1* | 0.26 | 4.96E-03 | *Protein Prenyltransferase Alpha Subunit Repeat Containing 1* – Regulates protein prenyltransferase activity | Protein prenylation |
| | *Cfap69* | 0.12 | 2.24E-02 | *Cilia And Flagella Associated Protein 69* – regulates sperm motility and sensory transduction | Flagillated motility |
| | **Trip13* | -0.14 | 8.88E-02 | *Thyroid Hormone Receptor Interactor 13* – required for chromosome recombination and chromosome structure development | Chromosome recombination |
| Inflammation | **Mkrn2* | 0.51 | 4.20E-03 | *Makorin Ring Finger Protein 2* – E3 ubiquitin ligase; regulates inflammatory responses | Protein ubiquitination |
| | *C1d* | -0.13 | 4.44E-04 | *C1D Nuclear Receptor Corepressor* – induces P53-dependent apoptosis; acts as a corepressor | rRNA processing & DNA repair |
| | *Cdca7* | 0.12 | 9.88E-02 | *Cell Division Cycle Associated 7* – regulates cell transformation, proliferation, and apoptosis | Cell cycle regulation & apoptosis |

B.

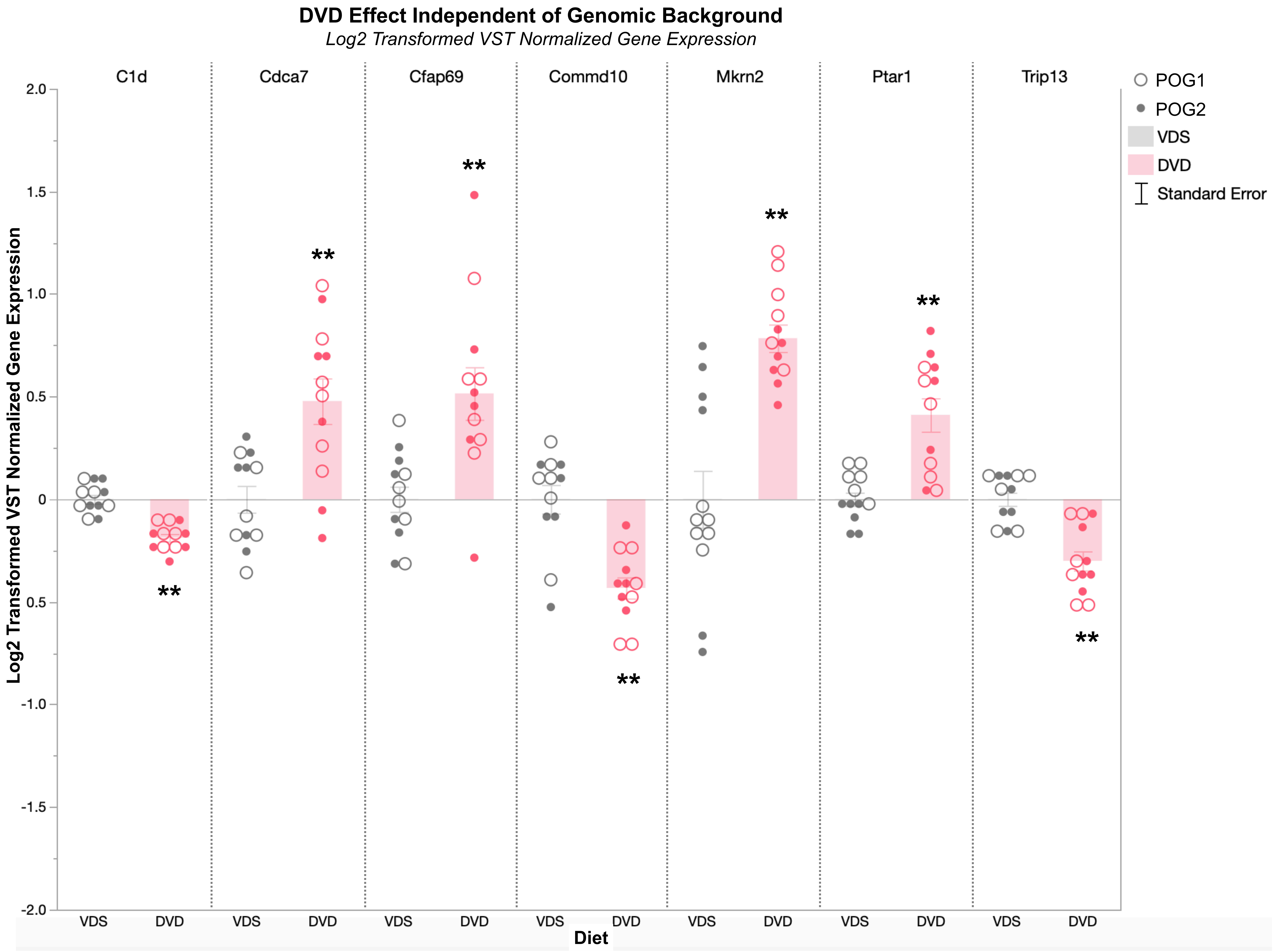


**Supplemental Figure 2. Diet Effects Independent of POG. (A)** DEGs with FDR<0.1 after adjustment for POG. Blue indicates downregulated by DVD. Red indicates upregulated by DVD. Single asterisk (*) indicates genes transcriptionally regulated by VDR. **(B)** Log2 transformed VST-normalized gene expression values for genes differentially expressed in POG1 (open circles) and POG2 (closed circles). Double asterisks (**) indicate FDR<0.1.

**Supplemental Figure 3. POG 1 Diet Signature.**

**A.**

| Gene Pathway Classification | Gene Name | Log2 Fold Change (DVD/VDS) | FDR | Gene Function (GeneCards (RRID:SCR_002773)) | Major Biological Process |
|---|---|---|---|---|---|
| Energy Metabolism | *Amy2a5* | 0.31 | 9.71E-02 | *Amylase Alpha 2A5* – catalyst for metabolism | Digestion & absorption |
| | *Nfya* | 0.09 | 8.07E-02 | *Nuclear Transcription Factor Y Subunit Alpha* – activator and repressor; regulates lipid metabolism | Steroid metabolism & transcription |
| Growth & Development | ***Commd10* | -0.09 | 9.71E-02 | *COMM Domain Containing 10* – regulates transcription and protein trafficking | Protein trafficking |
| | *Dmbt1* | 4.01 | 1.23E-03 | *Deleted In Malignant Brain Tumors 1* – tumor suppressor, plays a role in liver regeneration | Protein metabolism |
| | *Rab30* | 0.45 | 2.02E-02 | *RAB30, Member RAS Oncogene Family* – oncogene, regulates membrane trafficking | Membrane trafficking, protein metabolism & modification |
| | *Syt12* | 0.14 | 9.71E-02 | *Synaptotagmin 12* – regulates membrane trafficking | Membrane trafficking |
| | *Zbed4* | 0.09 | 9.71E-02 | *Zinc Finger BED-Type Containing 4* – regulates transcription | Transcription |
| Inflammation | **/**Mkrn2* | 1.12 | 3.98E-05 | *Makorin Ring Finger Protein 2* – E3 ubiquitin ligase; regulates inflammatory responses | Protein ubiquitination |
| | *Saa2* | 2.06 | 4.31E-03 | *Serum Amyloid A2* – regulates cholesterol homeostasis; highly expressed in response to inflammation | Acute-phase inflammatory response |

**B.**

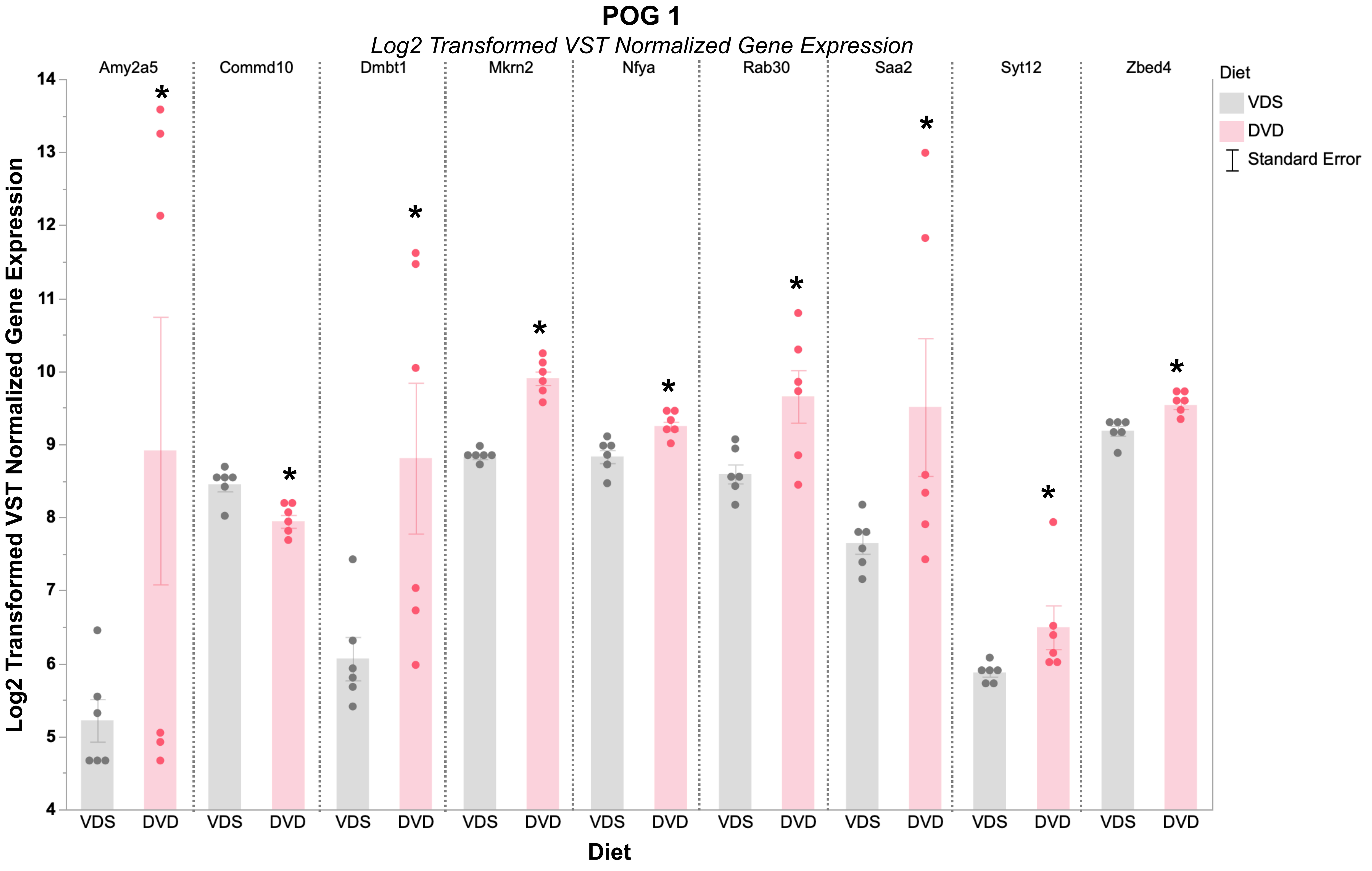


**C.**

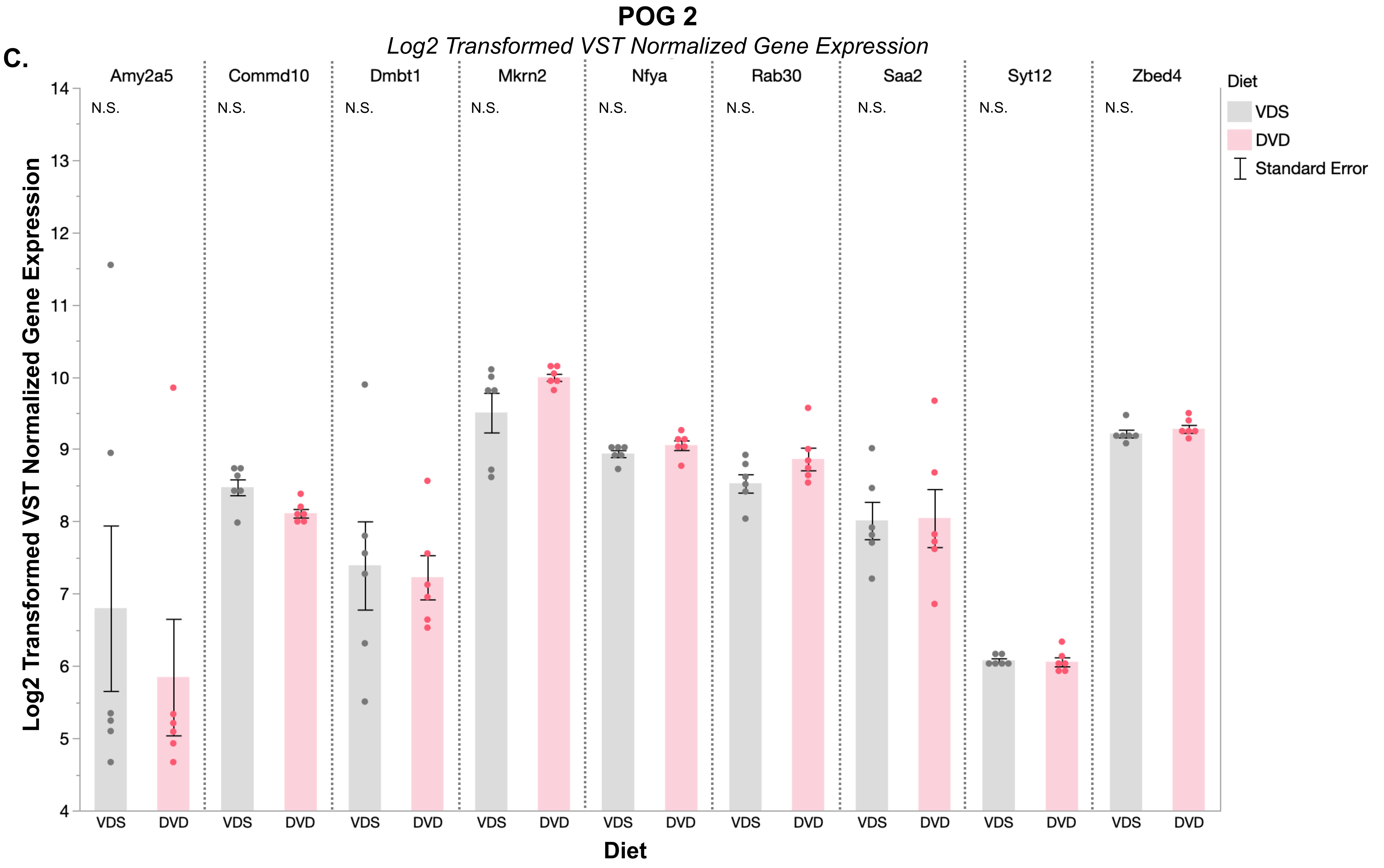


**Supplemental Figure 3. POG1 Diet Signature. (A)** Significantly differentially expressed genes with FDR<0.1 for POG1. Blue indicates downregulation by DVD. Red indicates upregulation by DVD. Double asterisks (**) indicate that a gene was previously identified in the diet adjusted for POG dataset. **(B)** Log2 transformed VST-normalized gene expression for POG1. Single asterisk (*) indicates FDR<0.1. **(C)** Log2 transformed VST-normalized gene expression values for POG2. N.S. = Not Significant.

**Supplemental Figure 4. POG 2 Diet Signature.**

A.

| Gene Pathway Classification | Gene Name | Log2 Fold Change (DVD/VDS) | FDR | Gene Function (GeneCards (RRID:SCR_002773)) | Major Biological Process |
|---|---|---|---|---|---|
| Cholesterol Biosynthesis | ***Ptar1* | 0.49 | 2.47E-03 | *Protein Prenyltransferase Alpha Subunit Repeat Containing 1* - regulates protein prenyltransferase activity | Protein prenylation |
| Energy Metabolism | *Slc7a15* | 1.09 | 1.39E-02 | *Solute Carrier Family 15 Member 5* – amino acid transporter | Amino acid transport |

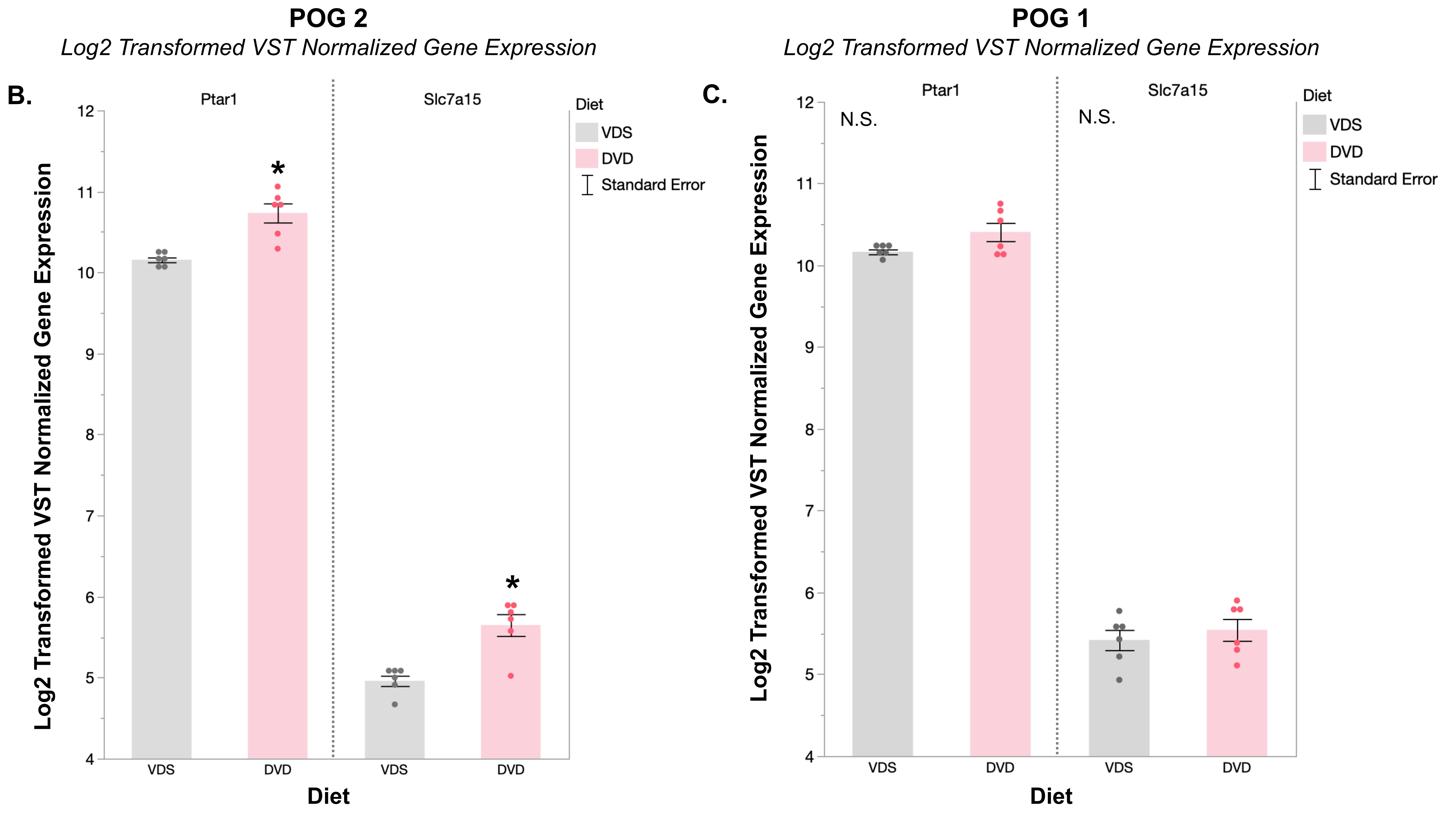


**Supplemental Figure 4. POG2 Diet Signature. (A)** Significantly differentially expressed genes with FDR<0.1 for POG2. Blue indicates downregulation by DVD. Red indicates upregulation by DVD. Double asterisks (**) indicate that a gene was previously identified in the diet adjusted for POG dataset. **(B)** Log2 transformed VST-normalized gene expression values for POG2. Single asterisk (*) indicates FDR<0.1. **(C)** Log2 transformed VST-normalized gene expression values for POG1. N.S. = Not Significant.

**Supplemental Figure 5. Fold change (DVD/VDS) of the 94 metabolites identified with significant (p<0.05) diet x POG interactions.**

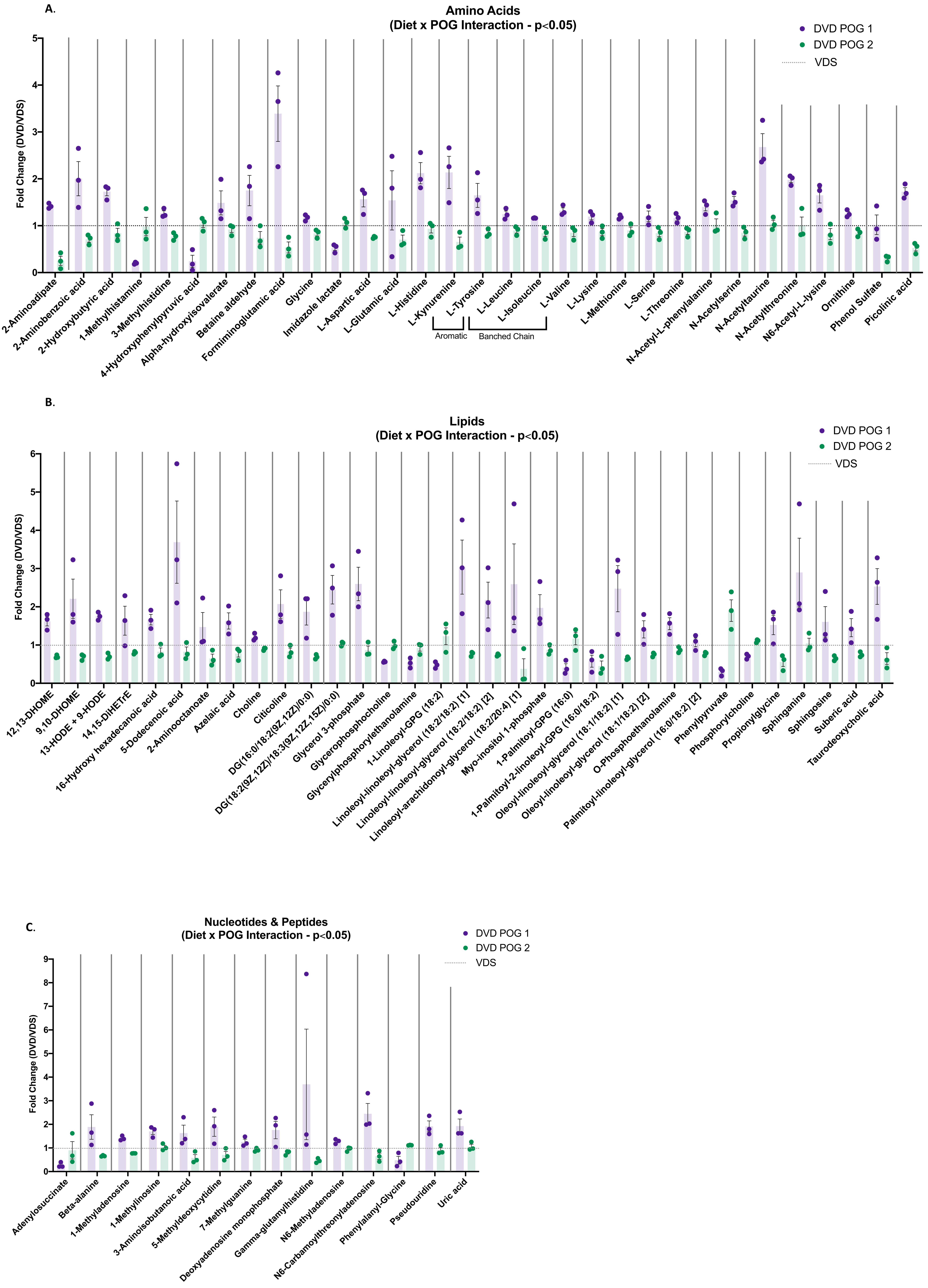

**Supplemental Figure 5**. **Fold change (DVD/VDS) of the 94 metabolites identified with significant (p<0.05) diet x POG interactions**.

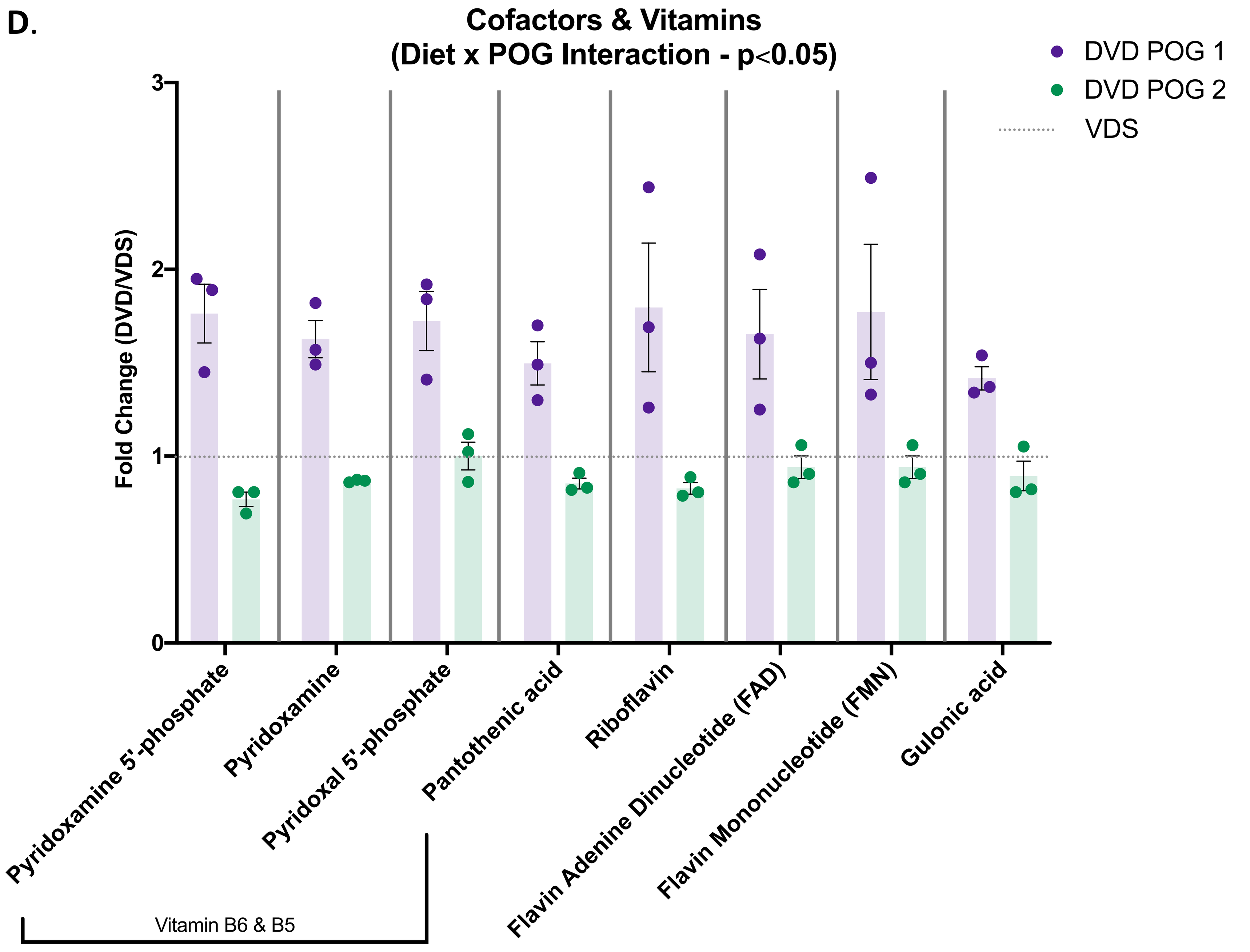


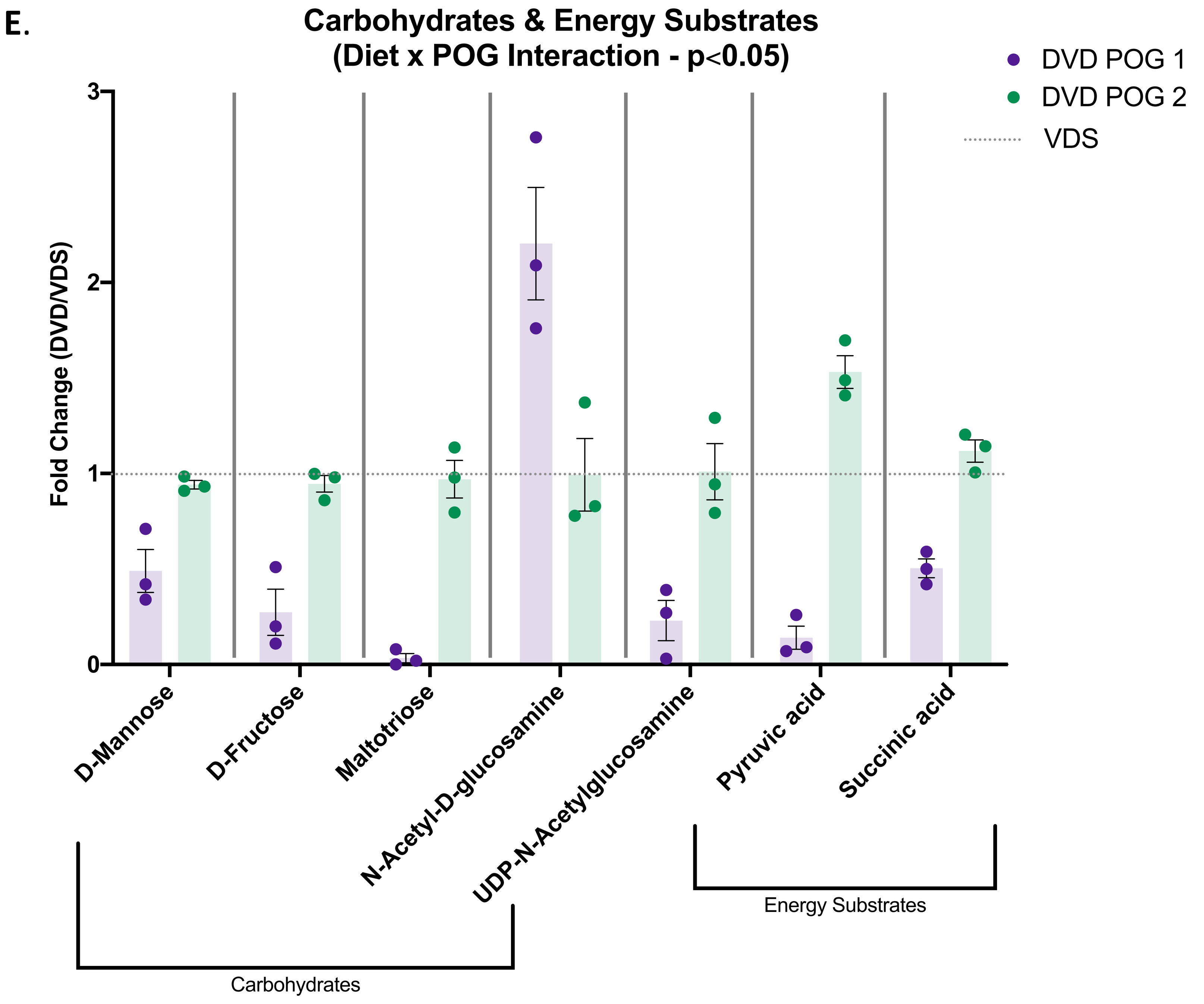


**Supplemental Figure 5**. **Fold change (DVD/VDS) of the 94 metabolites identified with significant diet x POG interactions with p<0.05. (A-E)** X-axis values >1 mean DVD increased abundance, while values <1 mean DVD decreased abundance.

**Supplemental Figure 6**. **DVD effects on methylated DMCs, genes, and gene overlap**.

**A.**

| Gene Pathway Classification | Gene Name | Gene Function (GeneCards (RRID:SCR_002773)) | Major Biological Process |
|---|---|---|---|
| Growth & Development | *Prrc2b* | *Proline Rich Coiled-Coil 2B* – Predicted to be involved in cell differentiation and to act upstream of or within embryonic development | Cell Differentiation |

**B.**

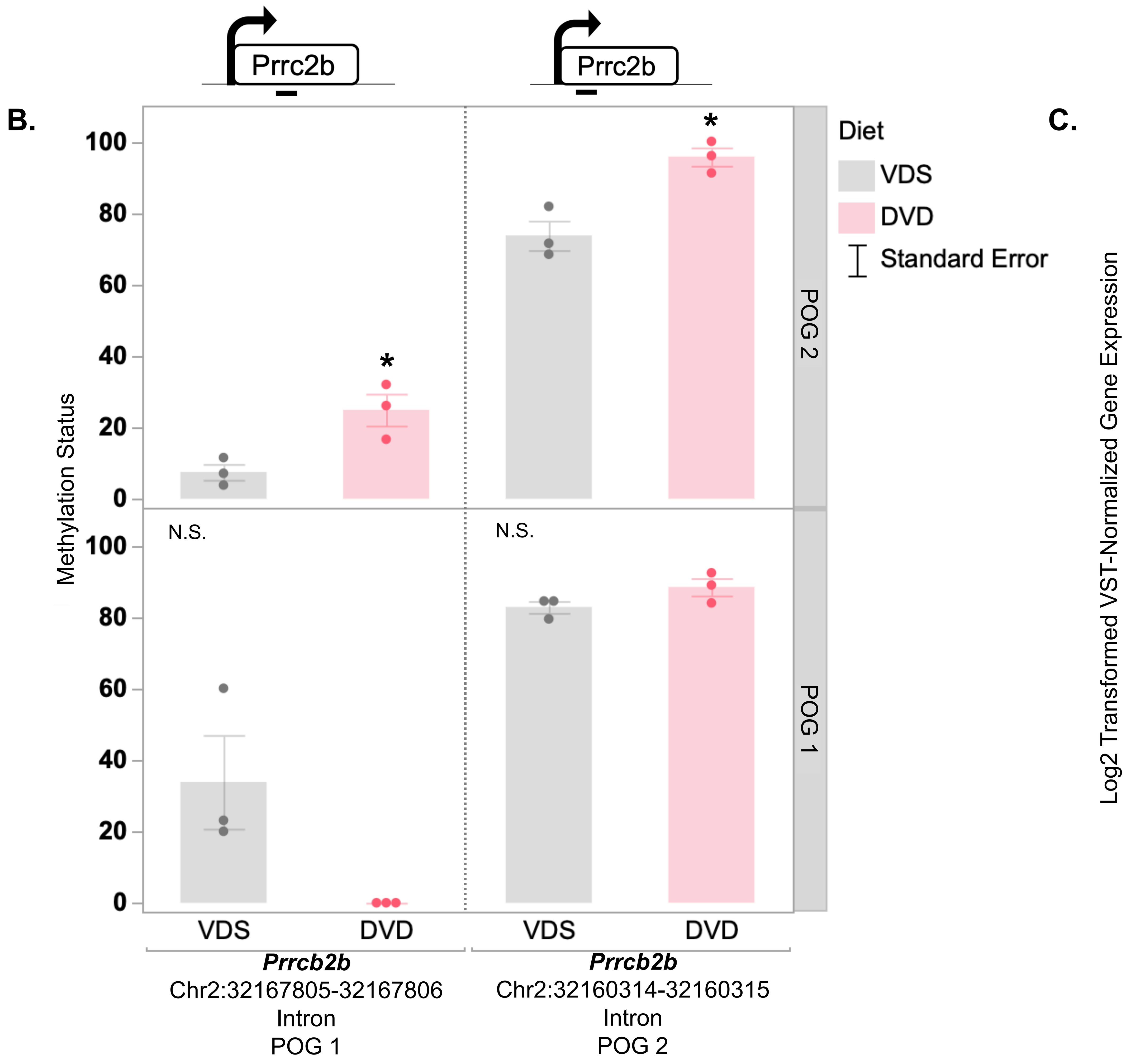


**C.**

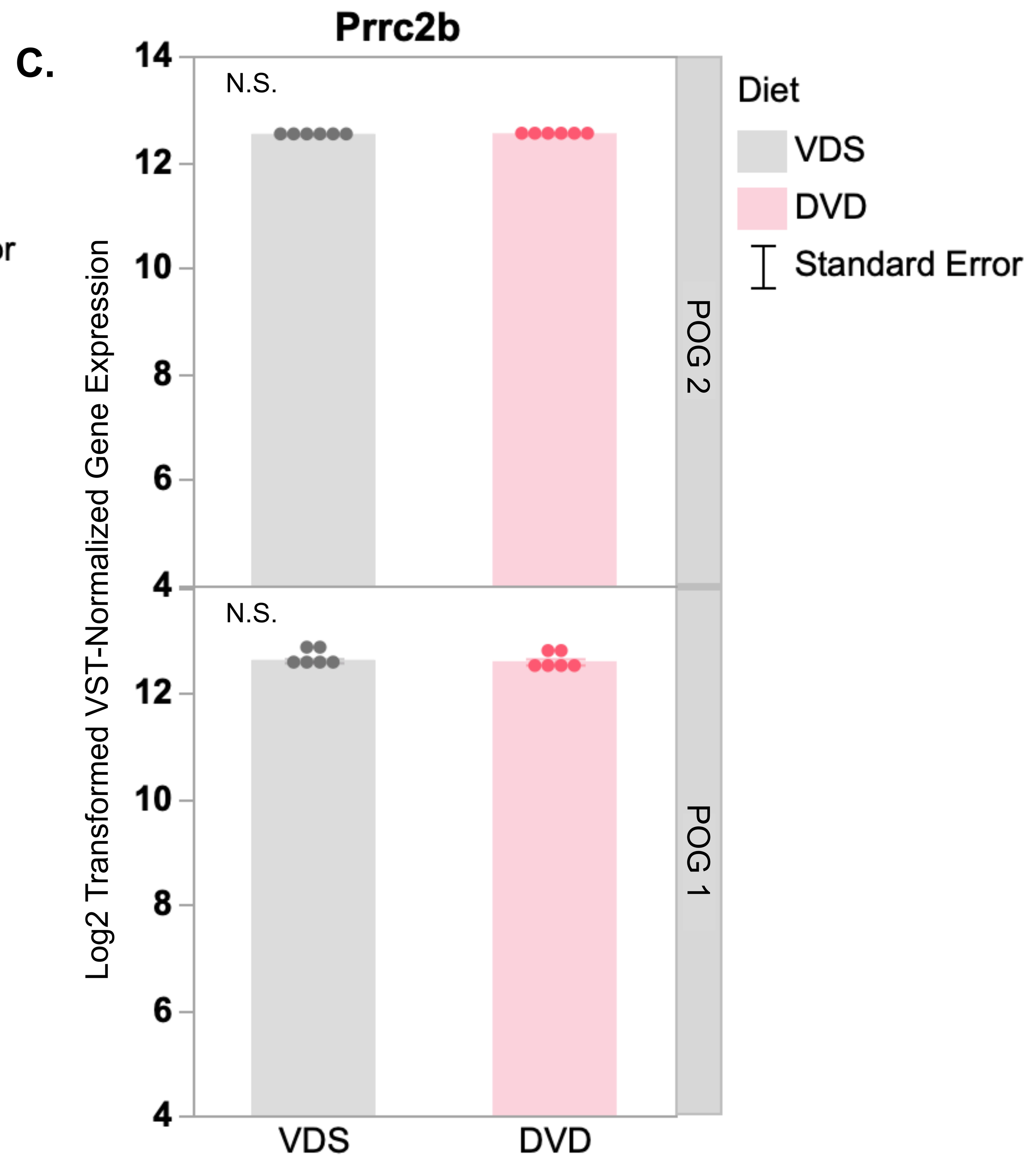


**Supplemental Figure 6**. **DVD effects on methylated DMCs, genes, and gene overlap**. **(A)** Description of overlapping DMG between POG1 and POG2. **(B)** Methylation status for POG1 and POG2. Single asterisk (*) indicates q<0.05. **(C)** Log2 transformed VST-normalized gene expression for overlapping gene on POG1 and POG2. N.S. = Not Significant.

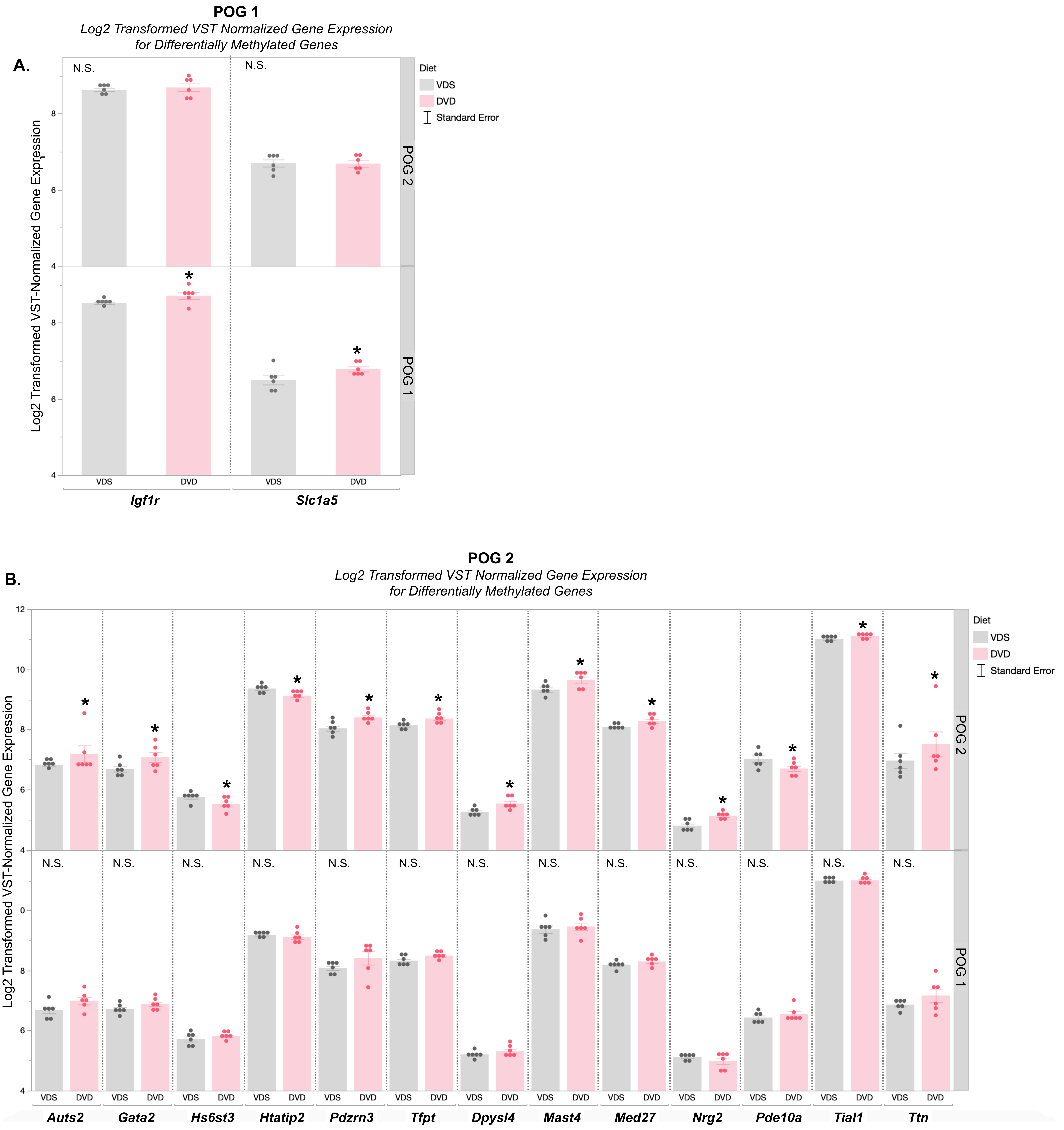


**Supplemental Figure 7. Gene expression for POG-specific differentially expressed and methylated genes. (A)** Log2 transformed VST-normalized gene expression for POG1 genes overlapping both RNA-Seq ($p<0.05$) and Bisulfite-Seq ($q<0.05$) datasets. **(B)** Log2 transformed VST-normalized gene expression for POG2 genes overlapping both RNA-Seq ($p<0.05$) and Bisulfite-Seq ($q<0.05$) datasets. Single asterisk (*) indicates significant changes for RNA-Seq ($p<0.05$) and Bisulfite-Seq ($q<0.05$) datasets. N.S. = Not Significant.

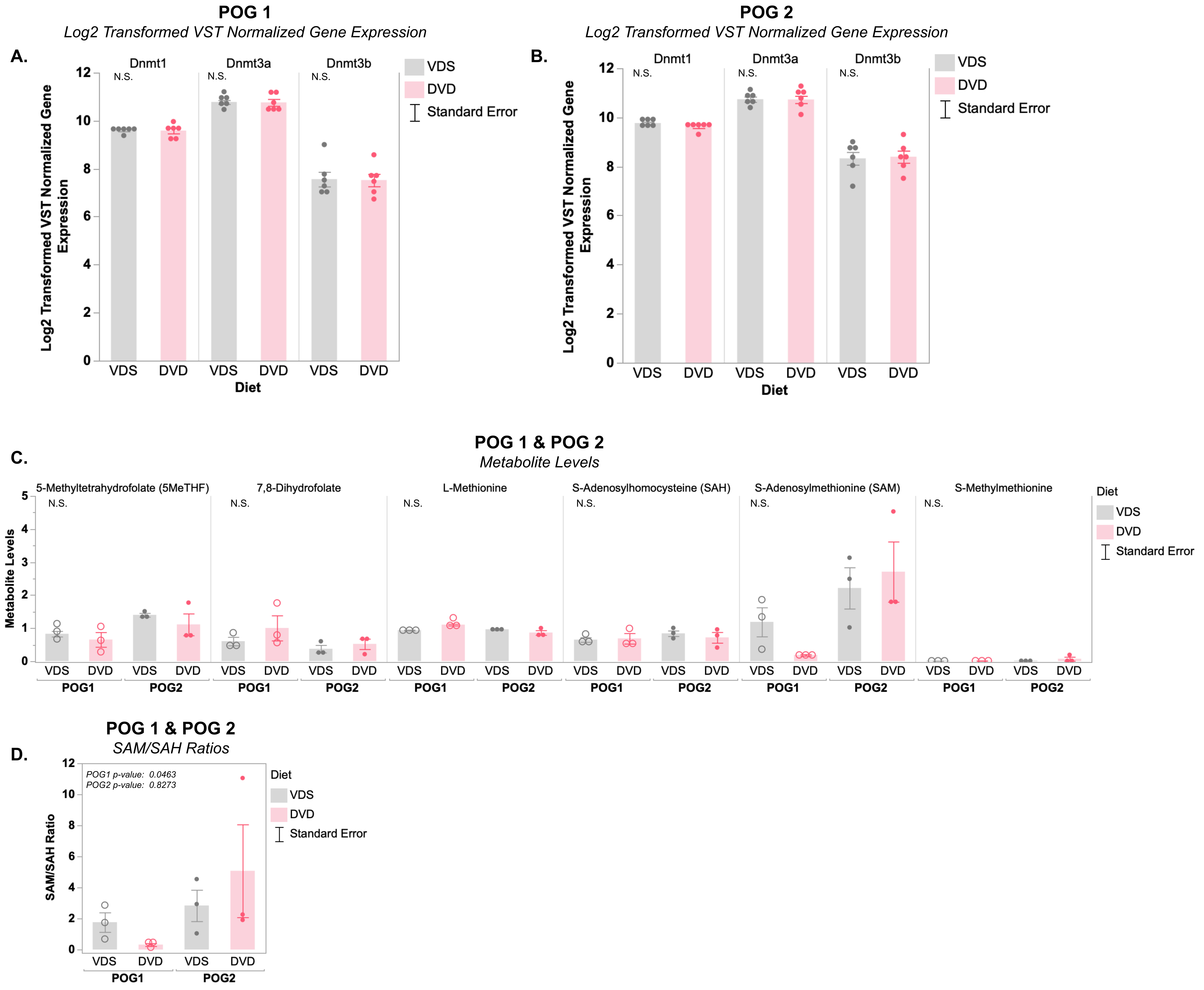


**Supplemental Figure 8. Gene expression and metabolite levels with known roles in DNA methylation. (A)** Log2 transformed VST-normalized gene expression for DNA methyltransferases on POG1. **(B)** Log2 transformed VST-normalized gene expression for DNA methyltransferases on POG2. **(C)** Levels of metabolites relevant to DNA methylation on POG1 and POG2. **(D)** SAM/SAH metabolite ratios for POG1 and POG2. P-values represent diet effects determined by Kruskal-Wallis test stratified by POG. N.S. = Not Significant.
