# Supplementary material for: Early life exposure to vitamin D deficiency impairs molecular mechanisms that regulate liver cholesterol biosynthesis, energy metabolism, inflammation, and detoxification
Source: Front Endocrinol (Lausanne). 2024 May 10;15:1335855. doi: 10.3389/fendo.2024.1335855 (PMC11116800; doi:10.3389/fendo.2024.1335855)
Supplement: Supplementary file 3 [file DataSheet_3.docx]

**Early life exposure to vitamin D deficiency impairs molecular mechanisms that regulate liver cholesterol biosynthesis, energy metabolism, inflammation, and detoxification**

**Authors:**

Megan M. Knuth^1,2^, Jing Xue^1,3^, Marwa Elnagheeb^1^, Raad Z. Gharaibeh^4,5^, Sarah A. Schoenrock^1^, Susan McRitchie^3^, Cory Brouwer^7,8^, Susan J. Sumner^3,6^, Lisa Tarantino^1,9,10^, William Valdar^1,2^, R. Scott Rector^11,12,13,14^, Jeremy Simon^1,15^, *Folami Ideraabdullah^1,2,3,6^

**Affiliations**:

^1^Department of Genetics, School of Medicine, University of North Carolina at Chapel Hill, Chapel Hill, NC 27599, USA;

^2^Lineberger Comprehensive Cancer Center, University of North Carolina at Chapel Hill, Chapel Hill, NC 27599, USA;

^3^Nutrition Research Institute, University of North Carolina at Chapel Hill, Kannapolis, NC 28081, USA;

^4^Department of Medicine, Division of Gastroenterology, University of Florida, Gainesville, FL 32608, USA;

^5^Department of Molecular Genetics and Microbiology, University of Florida, Gainesville, FL, USA;

^6^Department of Nutrition, Gillings School of Public Health, University of North Carolina at Chapel Hill, Chapel Hill, NC 27599, USA;

^7^Department of Bioinformatics and Genomics, University of North Carolina at Charlotte, Charlotte, NC 28223, USA;

^8^UNC Charlotte Bioinformatics Service Division, North Carolina Research Campus, Kannapolis, NC 28081, USA;

^9^Division of Pharmacotherapy and Experimental Therapeutics, Eshelman School of Pharmacy, University of North Carolina at Chapel Hill, Chapel Hill, NC 27599, USA;

^10^Department of Psychiatry, School of Medicine, University of North Carolina at Chapel Hill, Chapel Hill, NC, USA;

^11^Research Service, Harry S Truman Memorial Veterans Medical Center, Columbia, MO 65212, USA;

^12^NextGen Precision Health, University of Missouri, Columbia, MO 65211, USA;

^13^Department of Nutrition and Exercise Physiology, University of Missouri, Columbia, MO 65211, USA;

^14^Division of Gastroenterology and Hepatology, Department of Medicine, University of Missouri, Columbia, MO 65211, USA;

^15^Neuroscience Center Bioinformatics Core, University of North Carolina at Chapel Hill, Chapel Hill, NC 27599, USA.

**Corresponding Author**:

Folami Ideraabdullah

[folami@email.unc.edu](mailto:folami@email.unc.edu)

**Supplemental Table 2.** Genes within significantly overrepresented pathways dysregulated by DVD independent of POG.

| **Pathway Classification** | **Pathway** | **Gene Hits** | **Log2 Fold Change (DVD/VDS)** | **P-value** | **FDR** | **Gene Function (GeneCards (RRID:SCR_002773))** |
| --- | --- | --- | --- | --- | --- | --- |
| Cholesterol Biosynthesis | **Cholesterol Biosynthesis**  **(P00014)** | *Fdft1* | -0.06 | 3.25E-04 | 2.13E-01 | *Farnesyl-Diphosphate Farnesyltransferase 1* – first specific enzyme in liver cholesterol biosynthesis |
|  |  | *Mvk* | -0.05 | 9.62E-04 | 3.06E-01 | *Mevalonate kinase* – key early enzyme in isoprenoid and sterol synthesis |
|  |  | *Pmvk* | -0.04 | 1.83E-03 | 3.47E-01 | *Phosphomevalonate Kinase* – catalyzes the conversion of mevalonate 5-phosphate to mevalonate 5-diphosphate |
|  |  | *Fdps* | -0.03 | 2.14E-03 | 3.56E-01 | *Farnesyl Diphosphate Synthase* – key intermediate in liver cholesterol and sterol biosynthesis |
|  |  | *Idi1* | -0.02 | 9.05E-03 | 4.98E-01 | *Isopentenyl-Diphosphate Delta Isomerase 1* – catalyzes the interconversion of isopentenyl diphosphate to dimethylallyl diphosphate |
|  |  | *Hmgcr* | -0.03 | 2.12E-02 | 6.00E-01 | *3-Hydroxy-3-Methylglutaryl-CoA Reductase* – rate-limiting enzyme for cholesterol biosynthesis |
|  |  | *Lss* | -0.02 | 2.57E-02 | 6.14E-01 | *Lanosterol Synthase* – catalyst for the biosynthesis of cholesterol, steroid hormones, and vitamin D |
| Energy Metabolism | **Pentose Phosphate**  **(P02762)** | *Pgd* | -0.04 | 7.14E-03 | 4.70E-01 | *Phosphogluconate Dehydrogenase* – oxidative enzyme for the pentose phosphate pathway |
|  |  | *Tkt* | -0.06 | 6.83E-03 | 4.70E-01 | *Transketolase* – channels excess phosphate |
|  |  | *Hk2* | 0.03 | 1.09E-02 | 5.25E-01 | *Hexokinase 2* – phosphorylates glucose |
|  |  | *Taldo1* | -0.05 | 3.08E-02 | 6.29E-01 | *Transaldolase 1* – rate limiting non-oxidative enzyme and catalyst for the pentose phosphate pathway |
|  |  | *Fggy* | -0.04 | 3.77E-02 | 6.33E-01 | *FGGY Carbohydrate Kinase Domain Containing* – phosphorylates carbohydrates |
| Inflammation | **Parkinson’s Disease**  **(P00049)** | *Csnk2a2* | 0.04 | 4.29E-02 | 6.53E-01 | *Casein Kinase 2 Alpha* – phosphorylates acidic proteins |
|  |  | *Mapk3* | -0.06 | 9.37E-03 | 5.03E-01 | *Mitogen-Activated Protein Kinase 3* – extracellular signal-regulated kinase |
|  |  | *Mcm5* | -0.02 | 3.77E-02 | 6.33E-01 | *Minichromosome Maintenance Complex Component 5* – involved in the initiation of DNA replication |
|  |  | *Psma1* | -0.04 | 3.71E-02 | 6.33E-01 | *Proteasome 20S Subunit Alpha 1* – cleave peptides in an ATP/ubiquitin-dependent process |
|  |  | *Psma2* | -0.04 | 3.81E-02 | 6.33E-01 | *Proteasome 20S Subunit Alpha 2* – cleave peptides in an ATP/ubiquitin-dependent process |
|  |  | *Psma3* | -0.05 | 1.60E-02 | 5.50E-01 | *Proteasome 20S Subunit Alpha 3* – cleave peptides in an ATP/ubiquitin-dependent process |
|  |  | *Psma4* | -0.05 | 1.16E-02 | 5.29E-01 | *Proteasome 20S Subunit Alpha 4* – cleave peptides in an ATP/ubiquitin-dependent process |
|  |  | *Psma5* | -0.04 | 4.22E-02 | 6.51E-01 | *Proteasome 20S Subunit Alpha 5* – cleave peptides in an ATP/ubiquitin-dependent process |
|  |  | *Psma7* | -0.08 | 4.41E-04 | 2.30E-01 | *Proteasome 20S Subunit Alpha 7* – cleave peptides in an ATP/ubiquitin-dependent process |
|  |  | *Psmb1* | -0.05 | 3.71E-02 | 6.33E-01 | *Proteasome 20S Subunit Beta 1* – cleave peptides in an ATP/ubiquitin-dependent process |
|  |  | *Psmb3* | -0.07 | 9.53E-04 | 3.06E-01 | *Proteasome 20S Subunit Beta 3* – cleave peptides in an ATP/ubiquitin-dependent process |
|  |  | *Psmb4* | -0.06 | 5.45E-03 | 4.42E-01 | *Proteasome 20S Subunit Beta 4* – cleave peptides in an ATP/ubiquitin-dependent process |
|  |  | *Psmb7* | -0.07 | 1.49E-03 | 3.43E-01 | *Proteasome 20S Subunit Beta 7* – cleave peptides in an ATP/ubiquitin-dependent process |
|  |  | *Stub1* | -0.06 | 1.14E-02 | 5.29E-01 | *STIP1 Homology And U-Box Containing Protein 1* – ubiquitin ligase/cochaperone |
|  |  | *Ywhae* | -0.05 | 3.60E-02 | 6.31E-01 | *Tyrosine 3-Monooxygenase/Tryptophan 5-Monooxygenase Activation Protein Epsilon* – mediates signal transduction by binding to phosphoserine-containing proteins |

PANTHER overrepresentation pathway analysis for 1,338 DEGs with p<0.05. Blue indicates downregulation by DVD. Red indicates upregulation by DVD.

**Supplemental Table 3.** Cholesterol biosynthesis genes significantly overrepresented on POG2.

| **Pathway Classification** | **Pathway** | **Gene Hits** | **Log2 Fold Change (DVD/VDS)** | **P-value** | **FDR** | **Gene Function (GeneCards (RRID:SCR_002773))** |
| --- | --- | --- | --- | --- | --- | --- |
| Cholesterol Biosynthesis | **Cholesterol Biosynthesis (P00014)** | *Fdft1* | -0.002 | 2.28E-03 | 1 | *Farnesyl-Diphosphate Farnesyltransferase 1* – first specific enzyme in liver cholesterol biosynthesis |
|  |  | *Mvk* | -0.0002 | 2.81E-02 | 1 | *Mevalonate kinase* – key early enzyme in isoprenoid and sterol synthesis |
|  |  | *Pmvk* | -0.0006 | 8.78E-03 | 1 | *Phosphomevalonate Kinase* – catalyzes the conversion of mevalonate 5-phosphate to mevalonate 5-diphosphate |
|  |  | *Fdps* | -0.0002 | 3.08E-02 | 1 | *Farnesyl Diphosphate Synthase* – key intermediate in liver cholesterol and sterol biosynthesis |
|  |  | *Idi1* | -0.0005 | 1.17E-02 | 1 | *Isopentenyl-Diphosphate Delta Isomerase 1* – catalyzes the interconversion of isopentenyl diphosphate to dimethylallyl diphosphate |
|  |  | *Hmgcr* | -0.0003 | 2.49E-02 | 1 | *3-Hydroxy-3-Methylglutaryl-CoA Reductase* – rate-limiting enzyme for cholesterol biosynthesis |

PANTHER overrepresentation pathway analysis for 543 DEGs in POG2 with p<0.05. Blue indicates downregulation by DVD.

**Supplemental Table 4**. Metabolites with significant interactive (diet x POG) effects after correction for multiple testing (FDR<0.1).

| **Metabolite Name** | **Metabolite Class** | **Pathway** | **FDR** |
| --- | --- | --- | --- |
| Pyruvic acid | Energy Substrate | Glycolysis, Gluconeogenesis, and Pyruvate Metabolism | 1.72E-02 |
| N1-Methyladenosine | Nucleotide | Purine Metabolism | 1.72E-02 |
| 13-HODE + 9-HODE | Lipid | Fatty Acid Metabolism | 1.72E-02 |
| Ornithine | Amino Acid | Urea cycle; Arginine and Proline Metabolism | 3.22E-02 |
| L-Glutamic acid | Amino Acid | Glutamate Metabolism | 5.14E-02 |
| 12,13-DHOME | Lipid | Fatty Acid Metabolism | 6.43E-02 |
| Pyridoxamine 5'-phosphate | Cofactor | Vitamin B6 Metabolism | 6.43E-02 |
| Oleoyl-linoleoyl-glycerol (18:1/18:2) [1] | Lipid | Diacylglycerol Metabolism | 7.42E-02 |
| Palmitoyl-linoleoyl-glycerol (16:0/18:2) [1] | Lipid | Diacylglycerol Metabolism | 7.42E-02 |
| Pyridoxamine | Cofactor | Vitamin B6 Metabolism | 7.42E-02 |
| L-Aspartic acid | Amino Acid | Alanine and Aspartate Metabolism | 7.42E-02 |
| N-Acetyltaurine | Amino Acid | Methionine, Cysteine, SAM and Taurine Metabolism | 7.42E-02 |
| N-Acetylthreonine | Amino Acid | Glycine, Serine and Threonine Metabolism | 7.42E-02 |
| Histidine | Amino Acid | Histidine Metabolism | 9.65E-02 |
| Formiminoglutamate | Amino Acid | Histidine Metabolism | 9.65E-02 |
| 1-Methylhistamine | Amino Acid | Histidine Metabolism | 9.65E-02 |

16 metabolites with significant interactive (diet x POG) effects (FDR<0.1) after correction for multiple testing.

**Supplemental Table 5.** Metabolic pathways enriched (FDR<0.1) when considering 94 metabolites with Diet x POG interactions with p<0.05.

| **Pathway** | **Total # of Metabolites in Pathway** | **Expected # of Hits** | **Observed # of Hits** | **P-value** | **FDR** | **Metabolites** |
| --- | --- | --- | --- | --- | --- | --- |
| Beta-Alanine Metabolism | 34 | 1.53 | 9 | 7.58E-06 | 7.43E-04 | Beta-alanine; L-Glutamic acid; L-Histidine; L-Aspartic acid; Pantothenic acid; 3-Methylhistidine; FAD; Pyridoxal 5'-phosphate; Flavin mononucleotide |
| Ammonia Recycling | 32 | 1.44 | 8 | 4.16E-05 | 2.04E-03 | Glycine; L-Glutamic acid; L-Histidine; L-Serine; L-Aspartic acid; Pyruvic acid; FAD; Pyridoxal 5'-phosphate |
| Histidine Metabolism | 43 | 1.93 | 8 | 3.94E-04 | 1.29E-02 | Beta-alanine; L-Glutamic acid; L-Histidine; 3-Methylhistidine; Formiminoglutamic acid; 1-Methylhistamine; FAD; Pyridoxal 5'-phosphate |
| Glycine & Serine Metabolism | 59 | 2.65 | 9 | 7.76E-04 | 1.90E-02 | Glycine; L-Glutamic acid; L-Threonine; L-Serine; Ornithine; Pyruvic acid; L-Methionine; FAD; Pyridoxal 5'-phosphate |
| Vitamin B6 Metabolism | 20 | 0.898 | 5 | 1.37E-03 | 2.69E-02 | FAD; Pyridoxamine; Pyridoxal 5'-phosphate; Flavin mononucleotide; Pyridoxamine 5'-phosphate |
| Arginine & Proline Metabolism | 53 | 2.38 | 8 | 1.71E-03 | 2.79E-02 | Glycine; L-Glutamic acid; L-Aspartic acid; Ornithine; Succinic acid; FAD; Pyridoxal 5'-phosphate; Flavin mononucleotide |
| Glutamate Metabolism | 49 | 2.2 | 7 | 4.74E-03 | 6.64E-02 | Glycine; L-Glutamic acid; L-Aspartic acid; Pyruvic acid; Succinic acid; FAD; Pyridoxal 5'-phosphate |
| Alanine Metabolism | 17 | 0.764 | 4 | 5.53E-03 | 6.67E-02 | Glycine; L-Glutamic acid; Pyruvic acid; Pyridoxal 5’-phosphate |
| Sphingolipid Metabolism | 40 | 1.8 | 6 | 7.10E-03 | 6.67E-02 | L-Serine; O-Phosphoethanolamine; Sphingosine; Sphinganine; Pyridoxal 5'-phosphate; Phosphorylcholine |
| Phospholipid Biosynthesis | 29 | 1.3 | 5 | 7.75E-03 | 6.67E-02 | Glycerylphosphorylethanolamine; Glycerol 3-phosphate; FAD; Citicoline; Phosphorylcholine |
| Urea Cycle | 29 | 1.3 | 5 | 7.75E-03 | 6.67E-02 | L-Glutamic acid; L-Aspartic acid; Ornithine; Pyruvic acid; Pyridoxal 5'-phosphate |
| Malate-Aspartate Shuttle | 10 | 0.449 | 3 | 8.16E-03 | 6.67E-02 | L-Glutamic acid; L-Aspartic acid; Pyridoxal 5'-phosphate |
| Propanoate Metabolism | 42 | 1.89 | 6 | 9.06E-03 | 6.83E-02 | 2-Hydroxybutyric acid; Beta-alanine; L-Glutamic acid; L-Valine; FAD; Pyridoxal 5'-phosphate |
| Methylhistidine Metabolism | 4 | 0.18 | 2 | 1.12E-02 | 7.83E-02 | L-Histidine; 3-Methylhistidine |
| Carnitine Synthesis | 22 | 0.988 | 4 | 1.44E-02 | 0.9.4E-02 | Glycine; L-Lysine; Succinic acid; Pyridoxal 5'-phosphate |

**Supplemental Table 6**. Pathways with DMGs represented on POG1.

| **Pathway Classification** | **Pathway Name** | **# of Annotated Genes** |
| --- | --- | --- |
| Energy  Metabolism | Ionotropic glutamate receptor pathway (P00037) | 1 |
|  | N-acetylglucosamine metabolism (P02756) | 1 |
| Growth & Development | Gonadotropin-releasing hormone receptor pathway (P06664) | 1 |
|  | Cadherin signaling pathway (P00012) | 1 |
|  | Insulin/IGF pathway-protein kinase B signaling cascade (P00033) | 1 |
|  | Insulin/IGF pathway-mitogen activated protein kinase kinase/MAP kinase cascade (P00032) | 1 |
|  | Wnt signaling pathway (P00057) | 2 |
| Inflammation | TGF-beta signaling pathway (P00052) | 1 |
| Other | O-antigen biosynthesis (P02757) | 1 |

PANTHER pathway analysis for 40 DMGs with q<0.05 on POG1.

**Supplemental Table 7**. Top 15 pathways with DMGs represented on POG2.

| **Pathway Classification** | **Pathway Name** | **# of Annotated Genes** |
| --- | --- | --- |
| Growth & Development | Wnt signaling pathway (P00057) | 13 |
|  | Cadherin signaling pathway (P00012) | 10 |
|  | FGF signaling pathway (P00021) | 6 |
|  | PDGF signaling pathway (P00047) | 6 |
|  | EGF receptor signaling pathway (P00018) | 5 |
|  | Gonadotropin-releasing hormone receptor pathway (P06664) | 5 |
|  | VEGF signaling pathway (P00056) | 4 |
|  | Endothelin signaling pathway (P00019) | 4 |
|  | Axon guidance mediated by semaphorins (P00007) | 2 |
| Inflammation | Angiogenesis (P00005) | 4 |
|  | CCKR signaling map (P06959) | 4 |
|  | Huntington disease (P00029) | 3 |
|  | T cell activation (P00053) | 3 |
|  | Axon guidance mediated by semaphorins (P00007) | 2 |
|  | Apoptosis signaling pathway (P00006) | 2 |

PANTHER pathway analysis for 260 DMGs with q<0.05 on POG2.

**Supplemental Table 8**. Genes within significantly overrepresented pathways represented for POG2.

| **Pathway Classification** | **Pathway** | **Gene Hits** | **Methylation Change (DVD/VDS)** | **Q-value** | **Gene Function (GeneCards (RRID:SCR_002773))** |
| --- | --- | --- | --- | --- | --- |
| Growth & Development | **Cadherin Signaling**  **(P00012)** | *Cdh22* | -16.67 | 0.016 | *Cadherin 22* –  Plays an important role in morphogenesis and tissue formation in neural and non-neural cells during development |
|  |  | *Ctnna3* | -25.14 | 0.005 | *Catenin Alpha 3* –  Plays a role in cell-cell adhesion in muscle cells |
|  |  | *Pcdha4* | -31.47 | 0.025 | *Protocadherin Alpha 4* – Plays a critical role in the establishment and function of specific cell-cell connections in the brain |
|  |  | *Pcdha12* | -16.67 | 0.028 | *Protocadherin Alpha* 12 – Plays a critical role in the establishment and function of specific cell-cell connections in the brain |
|  |  | *Pcdhb5* | -31.06 | 0.016 | *Protocadherin Beta* 5 –  Plays a critical role in the establishment and function of specific cell-cell neural connections |
|  |  | *Pcdhb7* | -32.8 | 0.019 | *Protocadherin Beta* 7 –  Plays a critical role in the establishment and function of specific cell-cell neural connections |
|  |  | *Pcdhgc4* | 35.07 | 0.043 | *Protocadherin Gamma-C4* – Plays a critical role in the establishment and function of specific cell-cell connections in the brain |
|  |  | *Pcdhb14* | -29.51 | 0.024 | *Protocadherin Beta 14* – Plays a critical role in the establishment and function of specific cell-cell neural connections |
|  |  | *Pcdhb16* | -31.14 | 0.016 | *Protocadherin Beta 16* – Plays a critical role in the establishment and function of specific cell-cell neural connections |
|  |  | *Pcdhb17* | -25.35 | 0.038 | *Protocadherin Beta 17* – Plays a critical role in the establishment and function of specific cell-cell neural connections |
|  | **Wnt Signaling**  **(P00057)** | *Cdh22* | -16.67 | 0.016 | *Cadherin 22* –  Plays an important role in morphogenesis and tissue formation in neural and non-neural cells during development |
|  |  | *Csnk1d* | -15.12 | 0.045 | *Casein Kinase 1 Delta* – Implicated in the control of cytoplasmic and nuclear processes, including DNA replication and repair |
|  |  | *Ctbp2* | -34.06 | 0.018 | *C-Terminal Binding Protein 2* – Plays a role in chromatin binding and  transcription corepression |
|  |  | *Ctnna3* | -25.14 | 0.005 | *Catenin Alpha 3* –  Plays a role in cell-cell adhesion in muscle cells |
|  |  | *Pcdha4* | -31.47 | 0.025 | *Protocadherin Alpha 4* – Plays a critical role in the establishment and function of specific cell-cell connections in the brain |
|  |  | *Pcdha12* | -16.67 | 0.028 | *Protocadherin Alpha* 12 – Plays a critical role in the establishment and function of specific cell-cell connections in the brain |
|  |  | *Pcdhb5* | -31.06 | 0.016 | *Protocadherin Beta* 5 –  Plays a critical role in the establishment and function of specific cell-cell neural connections |
|  |  | *Pcdhb7* | -32.8 | 0.019 | *Protocadherin Beta* 7 –  Plays a critical role in the establishment and function of specific cell-cell neural connections |
|  |  | *Pcdhgc4* | 35.07 | 0.043 | *Protocadherin Gamma-C4* – Plays a critical role in the establishment and function of specific cell-cell connections in the brain |
|  |  | *Pcdhb14* | -29.51 | 0.024 | *Protocadherin Beta 14* – Plays a critical role in the establishment and function of specific cell-cell neural connections |
|  |  | *Pcdhb16* | -31.14 | 0.016 | *Protocadherin Beta 16* – Plays a critical role in the establishment and function of specific cell-cell neural connections |
|  |  | *Pcdhb17* | -25.35 | 0.038 | *Protocadherin Beta 17* – Plays a critical role in the establishment and function of specific cell-cell neural connections |
|  |  | *Prkca* | -34.68 | 0.01 | *Protein Kinase C Alpha* – Plays roles in many different cellular processes, such as cell adhesion, cell transformation, cell cycle checkpoint, and cell volume control |

PANTHER overrepresentation pathway analysis for 260 differentially methylated genes with q<0.05. Blue indicates downregulation by DVD. Red indicates upregulation by DVD.

**Supplemental Table 9**. Differentially expressed and methylated genes for each POG.

| **Gene Pathway Classification** | **Gene Name** | **POG** | **Methyl-ation**  **Status** | **Methyla-tion Change (DVD/VDS)** | **Log2 Fold Change (DVD/**  **VDS)** | **Gene Function (GeneCards (RRID:SCR_002773))** | **Major Biological Processes** |
| --- | --- | --- | --- | --- | --- | --- | --- |
| Growth & Development | *Igf1r* | POG 1 | LOM | -28.27 | 0.036 | *Insulin Like Growth Factor 1 Receptor* –critical role in cell transformation events | Cell Transformat-ion |
|  | *Slc1a5* |  | LOM | -41.91 | 0.022 | *Solute Carrier Family 1 Member 5* – amino acid transporter | Amino Acid Transport |
|  | *Auts2* | POG 2 | GOM | 37.85 | 0.0003 | *Activator Of Transcription And Developmental Regulator* – Component of a PCR1-like complex implicated in neurodevelopment & as a candidate gene for neurological disorders | Neurodevelo-pment |
|  | *Dpysl4* |  | LOM | -31.04 | 0.0004 | *Dihydropyrimidinase Like 4* – Involved in nervous system development | Nervous System Development |
|  | *Gata2* |  | GOM | 30.69 | 0.0008 | *GATA Binding Protein 2* – Regulates gene transcription & plays an important role in hematopoietic development | Gene Transcription |
|  | *Hs6st3* |  | GOM | 24.68 | -0.0002 | *Heparan Sulfate 6-O-Sulfotransferase 3* – Modifies heparin sulfate for protein interactions | Protein Interaction |
|  | *Htatip2* |  | GOM | 28.9 | -0.0008 | *HIV-1 Tat Interactive Protein* – Oxidoreductase; enables protein serine/threonine kinase activity | Tumor Suppression |
|  | *Mast4* |  | LOM | -32.78 | 0.0002 | *Microtubule Associated Serine/Threonine Kinase Family Member 4* – gives the kinase the ability to determine its own scaffold to control the effects of their kinase activities | Signal Transduction |
|  | **Med27* |  | LOM | -22.6 | 0.0002 | *Mediator Complex Subunit 27* – Regulates the transcription of RNA polymerase II-dependent genes | Gene Transcription |
|  | *Nrg2* |  | LOM | -29.71 | 0.0004 | *Neuregulin 2* – Induces growth and differentiation of epithelial, neuronal, glial, & other cell types | Cell Growth & Differentiation |
|  | *Pde10a* |  | LOM | -33.85 | -0.0003 | *Phosphodiesterase 10A* – Regulates the intracellular concentration of cyclic nucleotides | Signal Transduction |
|  | *Pdzrn3* |  | GOM | 11.57 | 0.0002 | *PDZ Domain Containing Ring Finger 3* – Regulates the differentiation of adipocytes, osteoblasts, & myoblasts | Cell Differentiation |
|  | **Tfpt* |  | GOM | 15.25 | 0.0005 | *TCF3 Fusion Partner* – Enables DNA binding activity & protein kinase binding activity; member of the INO80 complex | Gene Transcription |
|  | *Tial1* |  | LOM | -18.02 | 0.0002 | *TIA1 Cytotoxic Granule Associated RNA Binding Protein* – Regulates gene translation | Gene Translation |
|  | *Ttn* |  | LOM | -29.85 | 0.0003 | *Titin* – Key component in the assembly & function of striated muscles; plays a role in chromosome condensation & segregation during mitosis | Chromosome assembly |

Single asterisk (*) indicates genes transcriptionally regulated by VDR. Blue coloring indicates downregulation by DVD. Red coloring indicates upregulation by DVD.
